# The Roles of Zinc Finger Proteins in Colorectal Cancer

**DOI:** 10.3390/ijms241210249

**Published:** 2023-06-16

**Authors:** Aishwarya S. Iyer, Mohammed Rifat Shaik, Jean-Pierre Raufman, Guofeng Xie

**Affiliations:** 1 Department of Biochemistry and Molecular Biology, University of Maryland School of Medicine, Baltimore, MD 21201, USA; 2 Department of Medicine, University of Maryland Medical Center Midtown Campus, Baltimore, MD 21201, USA; 3 Division of Gastroenterology and Hepatology, Department of Medicine, University of Maryland School of Medicine, Baltimore, MD 21201, USA; 4 VA Maryland Healthcare System, Baltimore, MD 21201, USA; 5 Marlene and Stewart Greenebaum Cancer Center, University of Maryland School of Medicine, Baltimore, MD 21201, USA

**Keywords:** zinc finger protein, colorectal cancer, transcription factor, oncogene, tumor suppressor

## Abstract

Despite colorectal cancer remaining a leading worldwide cause of cancer-related death, there remains a paucity of effective treatments for advanced disease. The molecular mechanisms underlying the development of colorectal cancer include altered cell signaling and cell cycle regulation that may result from epigenetic modifications of gene expression and function. Acting as important transcriptional regulators of normal biological processes, zinc finger proteins also play key roles in regulating the cellular mechanisms underlying colorectal neoplasia. These actions impact cell differentiation and proliferation, epithelial–mesenchymal transition, apoptosis, homeostasis, senescence, and maintenance of stemness. With the goal of highlighting promising points of therapeutic intervention, we review the oncogenic and tumor suppressor roles of zinc finger proteins with respect to colorectal cancer tumorigenesis and progression.

## 1. Zinc Finger Proteins Are Transcriptional Regulators in Colorectal Cancer (CRC)

With more than 1.9 million new cases of CRC worldwide in 2020 [1] and 150,000 new cases projected in the US in 2023 [2], CRC is the third most prevalent cancer and second leading cause of cancer-related death in the world [3]. Potential contributors to increased CRC risk include the increasing prevalence of aging populations, ingestion of so-called Western diets, obesity, and insufficient physical exercise—all clearly interrelated factors. For currently unknown reasons, there has also been a shift towards the development of colorectal cancer in younger people, i.e., those younger than age 50 years [2]. Prior colon adenomas, inflammatory bowel disease, and a family history of CRC, including hereditary disorders (e.g., familial adenomatous polyposis and Lynch syndrome), further increase CRC risk [4]. Various molecular mechanisms are implicated in the development of CRC, including altered cellular signaling pathways, epigenetic modifications, genomic instability, and metabolic dysfunction. The complexity of CRC pathogenesis is further confounded by extensive cross-talk between these and other mechanisms [5]. The observation that zinc finger proteins play a key role as transcriptional regulators in the development and progression of CRC prompted the current review.

**Figure 1:**
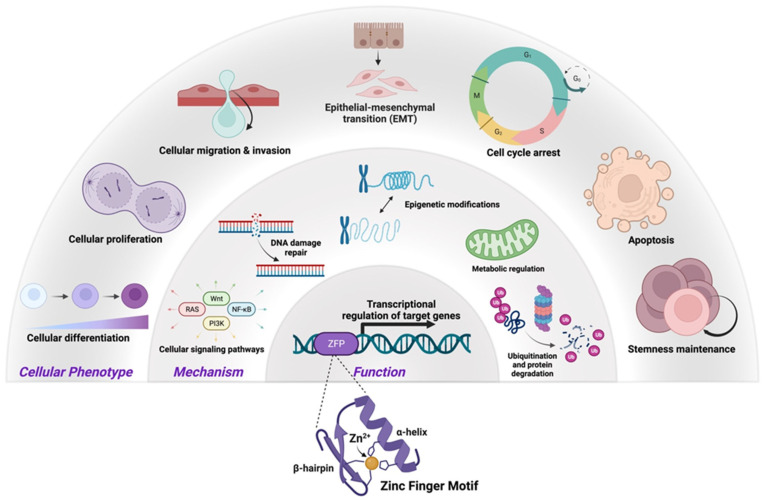
The roles of zinc finger proteins (ZFPs) in maintaining cellular homeostasis. ZFPs contain a zinc finger motif, vital for binding DNA and executing transcriptional regulation of target genes involved with mechanisms that maintain normal cellular physiology (i.e., signaling, DNA damage response, etc.). This figure illustrates the mechanisms whereby ZFP regulation of target gene transcriptional activity alters key cellular phenotypes. Created with Biorender.com.

For the purposes of this review, a zinc finger (ZNF) is defined as a small, functional, autonomously folded domain that requires the coordination of one or more zinc ions to stabilize its structure [6]. In 1985, xenopus transcription factor IIIA (TFIIIA) was the first protein shown to possess such a zinc finger domain [6]. ZNFs are DNA-binding domains that comprise the largest family of transcription factors. They can also bind RNA, facilitate interactions between proteins, have structural roles, and provide other, as-yet-undefined biological functions [6]. Hence, zinc finger proteins (ZFP) can act as transcriptional repressors and activators for a wide array of genes, thus modulating a multitude of biological processes, including cell differentiation, cell proliferation, migration, invasion, epithelial–mesenchymal transition (EMT), apoptosis, and stemness (Figure 1). As fundamental regulators of cancer progression via both oncogenic and tumor suppressor functions, ZFPs may also serve as therapeutic targets [7]. In this review, we discuss the roles of ZFPs in the development and progression of CRC, as either tumor suppressors or oncogenes, and highlight potential points of therapeutic intervention.

## 2. ZFPs Modulate CRC Cell Proliferation, Differentiation, Migration, Invasion, and EMT (Table 1)

Most ZFPs implicated in CRC progression are involved in manipulating the cellular phenotype to promote proliferative and invasive behavior and achieve metastatic potential through multiple mechanisms.

**Table 1:** ZFPs controlling cell proliferation, differentiation, migration, invasion, and EMT.

Molecular Mechanism	Gene	Common Alias(es)	Function	Additional Molecular Mechanisms	Cellular Targets	Role in Additional Cell Phenotypes or Behavior	Refs.
Wnt/β-catenin	*ZNRF3*	*RNF203*	TS		Frizzled, LRP6		[8,9]
	*OVOL2*	*CHED*; *CHED1*; *CHED2*; *PPCD1*; *ZNF339*	TS		TCF4, SLUG; MAP3K8/AKT/NF-κB signaling, CXCL16		[10,11]
	*FLYWCH1*		TS		β-catenin, TCF4, E-cadherin; γH2AX, ATM, p53	DNA damage response	[12,13]
	*ZFP36*	*Tristetraprolin*, *TIS11*, *TTP*, *NUP475*, *RNF162A*, *GOS24*	TS		*MACC1*, *ZEB1*, *SOX9*		[14]
	*RSPO2*	*HHRRD*; *TETAMS2*; *CRISTIN2*	TS		LGR5; Wnt5a/Fzd non-canonical pathway		[15,16]
	*ZIC2*	*HPE5*	O		*Axin2*; cyclin D1, CD44, LGR5	Apoptosis, cell cycle	[17]
	*ZBTB17*	*MIZ-1*, *ZNF151*, *ZNF60*, *pHZ-67*	O	Myc-signaling	Dpr1, Dvl2	Cell cycle	[18,19]
	*BMI1*	*FLVI2/BMI1*, *PCGF4*, *RNF51*	O		*p16^INK4a^*, *p14^ARF^*; *IDAX*, E-cadherin		[20,21,22,23]
	*PLAGL2*	*ZNF900*	O		ASCL2, Wnt4A, Wnt5A; Wnt6; CD44		[24,25,26]
	*ZNF281*	*GZP1*, *ZBP99*, *ZNP-99*	O			Cell cycle	[27]
	*GATA6*		O		Sp1, *urokinase-type plasminogen activator (uPA)*		[28]
	*TRIM37*	*MUL*, *POB1*, *TEF3*	O		β-catenin, c-Myc, cyclin D1		[29]
	*ZNF277*	*NRIF4*	O		β-catenin, p21^WAF1^	Cell senescence, cell cycle	[30,31]
	*ADNP*	*ADNP1*, *HVDAS*, *MRD28*	TS		Under investigation		[32]
Ras/ERK	*ZC3H13*	*Xio*	TS		Snail, cyclin D1, cyclin E1, occludin, Zo-1		[33]
	*ZDHHC9*	*DHHC9*, *MMSA1*, *MRZSR*, *MRXSZ*, *ZDHHC10*, *ZNF379*, *ZNF380*	O				[34]
JAK/STAT	*ZNF143*	*SBF*, *STAF*	TS		IL-8, *ZEB1*		[35,36]
	*ZNF460*	*HZF8*, *ZNF272*	O				[37]
NF-κB	*ZCCHC10*		TS				[38]
	*ZFP91*	*DSM8*, *FKSG11*, *PZF*, *ZNF757*	O		HIF-1α	Angiogenesis, cell cycle	[39]
PI3K/AKT	*ZNF549*		TS				[40]
	*GLI1*	*PAPA8*, *PPD1*	O	NF-κB		Stemness	[41,42]
	*ZNF692*	*AREBP*, *Zfp692*	O		cyclin D1, CDK2, MMP9, p27^Kip1^	Cell cycle	[43]
	*ZBED6*	*MGR*	TS	Hippo, TGF-β, EGFR, PI3K	IGF-2	Cell cycle	[44]
TGF-β	*ZNF37A*	*KOX21*, *ZNF37*	O		*THSD4*		[45]
	*ZNF326*	*ZAN75*, *ZIRD*	O		LTBP4, p-Smad2/3, N-cadherin, Snail, Slug, vimentin, E-cadherin, Zo-1		[46]
Hippo/YAP	*ZNF367*	*AFF29*, *CDC14B*, *ZFF29*	O				[47]
	*ZNF280A*	*SUHW1*, *ZNF280*, *ZNF636*	O			Cell cycle	[48]
YAP	*ZMYND8*	*PRKCBP1*, *PRO2893*, *RACK7*	O			Metabolism	[49,50]
DNA or histone methylation	*ZBTB18*	*MRD22*, *RP58*, *TAZ-1*, *ZNF238*	TS				[51]
	*GATA4*	*ASD2*, *TACHD*, *TOF*, *VSD1*	TS				[52]
	*GATA5*	*CHTD5*, *GATAS*					
	*ZNF677*		TS				[53]
	*ZFP82*	*ZNF545*	TS	Wnt/β-catenin, PI3K/AKT, MAPK/ERK, NF-κB, AP1 signaling	KAP1	Apoptosis, ribosome biogenesis	[54]
	*SPOP*	*BTBD32*, *TEF2*, *NSDVS1*	TS	Hh/GLI2 pathway	SP1/PI3K axis, HDAC6; MMP2	Apoptosis	[55,56,57]
	*PRDM5*	*BCS2*, *PFM2*	TS				[58]
	*UHRF1*	*ICBP90*, *Np95*, *RNF106*, *hNP95*, *TDRD22*	O				[59]
Epigenetic modifications	*MTA1*		O		VEGF-1, histone deacetylase	Angiogenesis	[60,61]
	*MORC2*	*CMT2Z*, *DIGFAN*, *ZCW3*, *ZCWCC1*	O		NDRG1		[62]
	*ZNF518B*		O		Histone methyltransferases		[63]
	*ZNF146*	*OZF*	O		hRAP		[64,65]
	*ZNF382*	*KS1*	TS		HP1, NF-κB, and AP1 signaling	Apoptosis	[66]
	*KLF5*	*BTEB2*, *CKLF*, *IKLF*	O		SNHG12	Stemness, DNA damage response, cell cycle	[67]
	*Sp1*		O		ZFAS1, VEGFA, miR-150-5p	Apoptosis	[68,69]
	*THAP11*	*RONIN*	O		HCF-1		[70]
	*ZBTB48*	*TZAP*, *HKR3*, *ZNF855*	O		Telomere dysregulation, mechanism under investigation		[71]
DNA Damage Response	*KLF4*	*EZF*, *GKLF*	TS	Wnt, ERK signaling	p53, histone acetylases; NDRG2, cyclin D1; u-PAR	Cell cycle	[72,73,74,75]
	*ZEB1*	*AREB6*, *BZP*, *DELTAEF1*, *FECD6*, *NIL2A*, *PPCD3*, *TCF8*, *ZFHEP*, *ZFHX1A*	O	Wnt pathway	MPG; LOXL2; uPA, PAI-1; miR-200	Stemness	[76,77,78,79,80]
	*HLTF*	*HIP116*, *HIP116A*, *HLTF1*, *RNF80*, *SMARCA3*, *SNF2L3*, *ZBU1*	TS				[81]
p53	*ZBTB7A*	*FBI1*, *LRF*, *ZBTB7*, *ZNF857A*, *pokemon*, *TIP21*	TS/O		ETS-1, MMPs; NF-κB	Glycolysis	[82,83,84,85]
	*GLI3*	*ACLS*, *GCPS*, *GLI3-190*, *GLI3FL*, *PAPA*, *PAPB*, *PHS*, *PPDIV*	O	ERK1/2 cascade			[86,87]
	*ZNF398*	*p51*, *p71*, *ZER6*	O		MDM2	Cell cycle	[88]
E-cadherin	*SNAI1*	*SNAH*, *SLUGH2*, *SNAIL1*, *SNAIL*	O	Wnt/β-catenin signaling	*VDR*; *lncRNA WiNTRLINC1*, *MYB*	Stemness	[89,90,91]
	*SPRY2*	*SPROUTY2*, *IGAN3*	O		ZEB1, E-cadherin		[92]
	*NANOS1*	*NOS1*, *SPGF12*, *ZC2HC12A*	O		p120ctn		[93]
	*ZC3H12C*	*MCPIP3*	TS		*Vimentin*, VCAM-1, *MMP2*		[94]
Metabolism	*YY1*	*DELTA*, *NF-E1*, *UCRBP*, *YIN-YANG-1*	O	Wnt/β-catenin signaling	GLUT3; p53	Cell cycle, apoptosis	[95,96]
	*ZBTB7C*	*APM1*, *ZBTB36*, *ZNF857C*	TS	Ras, Wnt signaling	Myc	Inflammation	[97,98]
	*ZFP1*	*ZNF475*, *PITA*	O		p53	Apoptosis	[99]
	*ZNF568*	*PISA*	O		p53	Apoptosis	[99]
Angiogenesis	*ZNF384*	*CAGH1*, *CAGH1A*, *CIZ*, *ERDA2*, *NMP4*, *NP*, *TNRC1*	O		MMP2		[100]
	*SNAI2*	*SLUG*, *SNAIL2*, *WS2D*, *SLUGH1*	O		MMPs; MDM2, p53/p21; *VDR*		[89,101,102,103,104]
	*ZEB2*	*SMADIP1*, *SIP1*, *ZFHX1B*	O	miR-192, E-cadherin	MMP-2/9	Metastasis, EMT	[105,106,107,108]
	*ZNF24*	*ZNF191*, *ZSCAN3*, *KOX17*	TS		VEGF		[109]
	*ZKSCAN3*	*ZF47*, *ZFP306*, *ZNF306*, *ZNF309*, *ZSCAN13*, *ZSCAN35*	O		VEGF, integrin β4		[110,111]
Under investigation	*WT1*	*AWT1*, *GUD*, *NPHS4*, *WAGR*, *WIT-2*, *WT33*	O				[112,113]
	ZBTB4	*Kaiso-L1*, *ZNF903*	TS				[114]
	*FOXP1*	*12CC4*, *HSPC215*, *MFH*, *QRF1*, *hFKH1B*	TS			Inflammation	[115,116]
	*ZNF185*	*SCELL*	O				[117]
	*ZNF217*	*ZABC1*	O				[118]
	*ZNF703*	*NLZ1*, *ZEPPO1*, *ZNF503L*, *ZPO1*	O				[119]
	*CRIP1*	*CRHP*, *CRIP*	O		GSK3/mTOR		[120,121]
	*ZFC3H1*	*CCDC131*, *CSRC2*, *PSRC2*	O				[122]
	*ZFR*	*SPG71*, *ZFR1*	O		FAM49B		[123]
	*ZNF350*	*ZBRK1*, *ZFQR*	TS		ATXN2		[124]

TS = tumor suppressor, O = oncogene; the NCBI Gene database was utilized to determine common alias(es).

### 2.1. ZFPs Modulate Wnt Signaling

The canonical Wnt pathway modulates β-catenin/T cell factor (TCF) transcription factor complex activity to regulate target gene expression and promote cell differentiation, proliferation, and migration. Briefly, after Wnt binding to its target receptor, comprising LRP5/6 and Frizzled (Fzd), Dishevelled (Dvl) is recruited to block cytosolic β-catenin phosphorylation and subsequent degradation by a destruction complex comprising glycogen synthase kinase 3β (GSK3β), axin, casein kinase I (CK1), and adenomatous polyposis coli (APC). β-catenin then undergoes nuclear translocation where it complexes with TCF to induce target gene expression [9]. Inhibition of this pathway is commonly regulated by **ZNRF3**/RNF43, an E3 ubiquitin ligase, that degrades FZD and LRP6 to promote β-catenin ubiquitination [8,9].

Non-canonical Wnt pathways encompassing cascades independent of the β-catenin/ TCF complex classically control cell migratory and polarity phenotypes; the two most common non-canonical pathways are the planar cell polarity and calcium-activated pathways [9,125].

Numerous ZFPs downregulate Wnt signaling by different mechanisms, thereby attenuating CRC cell motility and invasive behavior. This can occur by restricting the expression of EMT-promoting genes, e.g., blocking pro-EMT Wnt signaling via **OVOL2**-mediated recruitment of histone deacetylase to the TCF4–β-catenin complex [10]. **FLYWCH1** stimulates reduced cell motility and enhances cell attachment through β-catenin interactions and transcriptional modulation of β-catenin/TCF4 to impede the expression of downstream genes including ***ZEB1***, *EPHA4*, and *E-cadherin* [13]. **ZFP36**, negatively correlated with Wnt/β-catenin signaling activity, represses the expression of EMT-related transcription factors including *MACC1*, *ZEB1*, and *SOX9* [14]. While R-spondin proteins usually enhance Wnt signaling through **ZNRF3** turnover, **RSPO2** promotes the stabilization of membrane-associated ZNRF3 through LGR5-dependent interactions [15]. These interactions reduce CRC cell motility and proliferation, likely by inhibiting the Wnt5a-activated non-canonical pathway [16]; this highlights the complexity and interactions between signaling pathways underlying CRC pathogenesis.

Many other ZFPs function to augment Wnt signaling. **ZIC2** promotes transcriptional repression of *Axin2*, a vital component of the β-catenin destruction complex, and/or direct interactions with β-catenin [17]. **ZBTB17** downregulates Dapper1, a protein that degrades Dvl, thereby enhancing Wnt signaling [18], while **BMI1** suppresses *IDAX*, a CXXC-type zinc finger domain-containing protein and Wnt pathway repressor, to increase cell proliferation [22]. **PLAGL2** facilitates both canonical and non-canonical Wnt signaling by activating Wnt6 and ASCL2 to promote intestinal epithelial stem cell identity, and activating Wnt4a and Wnt5a, actions that promote enteroid growth [24,26]. Other ZFPs that augment Wnt signaling and promote cell proliferation and CRC metastatic potential include **ZNF281** [27], **GATA6** [28], and **TRIM37** [29].

**ZNF277**, a senescence-regulating transcription factor upregulated in CRC, is also a transcriptional target of β-catenin. Previous work by our group demonstrated that **ZNF277** mRNA and protein expression modulate key cancer pathways, including the HOXD family and p21^WAF1^, and play a key role in M_3_ muscarinic receptor-dependent murine CRC progression [30,31].

### 2.2. ZFPs Modulate Other Signaling Pathways

While Wnt signaling is a key modulator of colon cancer progression, many other pathways are also exploited by ZFPs. Notably, the Ras/ERK pathway, mutated in many cancers, is inhibited by **ZC3H13**, an anti-oncogene downregulated in CRC, thus attenuating cell proliferation and invasion [33]. Conversely, ***ZDHHC9***, which is upregulated in CRC, potentially modulates the N-Ras and H-Ras pathway via its palmitoyl transferase activity; the precise mechanism of how this promotes tumorgenicity remains obscure [34].

Activation of intracellular Janus kinases (JAKs) via ligand-mediated receptor activation leads to downstream phosphorylation and dimerization of STAT proteins; this STAT complex functions as a nuclear transcription factor that promotes the expression of genes beneficial for tumorigenesis [126]. For example, in addition to regulating the Ras/ERK pathway, **ZNF143** controls the JAK/STAT pathway to alter the expression of IL-8, a pro-angiogenic cytokine, thereby curtailing tumor progression [35]. **ZNF460** promotes CRC metastasis by stimulating JAK2/STAT3 signaling [37].

In the absence of an inhibitor, IκB, the NF-κB transcription factor, comprising p65 and p50 subunits, is translocated to the nucleus where it modulates the expression of target genes that promote cell proliferation, migration, and invasion [127]. Activation of NF-κB signaling can occur via transcriptional repression of **ZCCHC10** by miR-410-3p [38]. Alternatively, ZFPs such as **ZFP91** can interact with the p65 subunit of NF-κB to stimulate transcription of the alpha-subunit of the transcription factor hypoxia-inducible factor 1 (HIF-1α) to regulate processes that promote tumorigenesis, including cell proliferation, invasion, metastasis, angiogenesis, and cell cycle progression [39].

Phosphatidylinositide 3-kinase (PI3K) is activated downstream of many receptor types to phosphorylate cell membrane-adherent phosphoinositides. Following a series of subsequent recruitment and binding events, involving various enzyme complexes, the AKT kinase is ultimately turned on to induce mTOR, MDM2, and FOXO activation resulting in accelerated cell differentiation and proliferation [128]. miR-708-5p, an enhancer of PI3K/AKT signaling, negatively regulates **ZNF549** to induce CRC cell proliferation and migration [40]. Interestingly, while **ZNF549** expression in primary tumors is reduced compared to normal tissue, its expression is increased in advanced-stage tumors, suggesting additional roles for this ZFP in CRC progression [40]. Conversely, **GLI1**, a vital player in Hedgehog signaling, enhances PI3K/AKT signaling in a Foxm1-dependent manner to increase CRC metastasis [42], while **ZNF692** promotes cell proliferation and invasion by altering the expression of proteins involved in cell cycle regulation and angiogenesis, including cyclin D1, CDK2, and p27^Kip1^, via the PI3K/AKT pathway [43]. **ZBED6**, a repressor of IGF2 expression, attenuates cell proliferation, likely via PI3K signaling, and has also been implicated as a modulator of the Wnt, Hippo, TGF, and EGFR pathways [44].

TGF-β ligand–receptor binding results in the phosphorylation of cytosolic SMAD proteins and the formation of SMAD multimer complexes that are translocated to the nucleus and bind specific DNA motifs, thus regulating target gene expression [129]. Interestingly, the enhancement of TGF-β signaling by **ZNF37A**-induced repression of the tumor microenvironment regulator *THSD4* stimulates cytokine generation by cancer-associated fibroblasts to promote tumor spread [45]. TGF-β pathway activation to increase invasive potential can also occur via upregulation of **ZNF326** target genes, e.g., LTBP4, a TGF-β1 receptor activator [46]. Both **ZNF367** [47] and **ZNF280A** [48] inhibit the Hippo tumor suppressor pathway, augmenting Yes-associated protein (YAP) signaling to enhance CRC cell proliferation. **ZMYND8**, a multifunctional transcription factor, histone reader, and DNA repair protein, is also a target of YAP-mediated signaling that induces the cholesterol synthesis necessary for CRC cell proliferation [50].

### 2.3. ZFPs Modulate Epigenetic Modifications

Epigenetic modifications are broadly characterized by altered gene expression in the absence of DNA sequence changes. The most well studied modifications involved in CRC pathogenesis include altered target gene promoter methylation status and/or recruitment of histone modifiers (i.e., acetylators and methylators) to alter gene expression levels [130]. Non-coding RNAs such as long non-coding RNAs (lncRNAs) and microRNAs (miRNAs) have also emerged as epigenetic modulators [131]. Promoter methylation often inactivates or downregulates target tumor suppressor ZFPs, including **ZBTB18** [51], **GATA4/GATA5** [52], and **ZNF677** [53], to promote CRC pathogenesis via CRC cell proliferation and invasion. This can also occur via promoter hypermethylation, as seen with **ZFP82**, which normally interacts with the corepressor KAP1, to attenuate rRNA transcription and ribosome biogenesis via histone deacetylation [54], and **SPOP**, which facilities polyubiquitination and subsequent degradation of histone deacetylase 6 (HDAC6) to reduce deacetylation of non-histone proteins and prevent EMT [56,57]. Interestingly, **PRDM5**, a tumor suppressor underscored by its ability to inhibit cell proliferation after its transfection into human CRC cells, is also inactivated by trimethylation of histone H3 lysine [58]. Conversely, **UHRF1**, an E3 ubiquitin ligase, can initiate and maintain DNA hypermethylation to silence tumor suppressor genes and promote metastasis [59].

Interactions with histone deacetylases by ZFPs can also promote CRC metastasis. **MTA1**, whose expression is increased in CRC liver metastases, was shown to interact with histone deacetylase and modulate chromatin structure and transcriptional repression [61]. Similarly, **MORC2** interacts with the histone deacetylase Sirutin1 to suppress downstream Myc-regulated gene 1*N* (*NDRG1*) transcription, ultimately promoting EMT and CRC lung metastasis [62]. Alternatively, **ZNF518B** expression promotes CRC metastasis and recurrence by recruiting histone methyltransferases (e.g., EZH2 and G9A) to silence downstream tumor suppressor genes [63]. **ZNF146** enhances CRC cell proliferation and motility [65] by telomere dysregulation through interactions with hRAP, a telomeric protein that modulates telomere length [64]. Heterochromatin silencing via heterochromatin protein 1 interactions is a potential mechanism whereby **ZNF382** inhibits tumorigenesis by altering the expression of oncogenes and other factors involved in NF-κB signaling [66].

Epigenetic modifications of DNA or chromatin are not the only mechanisms utilized to induce CRC oncogenicity. **KLF5**, a downstream mediator of activated K-Ras and H-Ras, targets lncRNAs to modulate the expression of protein-coding genes via interactions with co-regulatory transcription factors, such as AR and HSF1 [67]. Similarly, **Sp1** induces expression of the lncRNA ZFAS1, which binds miR-150-5p, to upregulate VEGFA and subsequently promote cell proliferation, migration, and invasion [68,69]. Primary tumors and metastases overexpress **THAP11** which binds to host cell factor-1 (HCF-1), a transcriptional coregulator, to target promoters, ultimately modulating transcription and cell proliferation via histone modifications [70].

### 2.4. ZFPs and the DNA Damage Response

Evasion of DNA damage response pathways during cell growth and proliferation is important for CRC progression. Non-homologous end joining (NHEJ) and homologous recombination (HR) are the major double-strand DNA break repair pathways, the latter requiring proto-oncogenes BRAC1 and BRCA2 for successful repair [132]. Other types of DNA damage, such as single-stranded DNA mishaps, utilize mismatch repair (MMR) and base excision repair (BER) to repair or remove mismatched nucleotides [133]. Defects in any of these pathways contribute to genomic instability and the accumulation of mutations that promote cancer cell phenotypes. **ZEB1**, a well-studied ZFP, promotes colitis-associated CRC by repressing transcriptional repair molecules, including N-methyl-purine glycosylase, involved in the BER pathway. ZEB1 upregulation in CRC cells also stimulates macrophages to generate reactive oxygen species and IL-1β, which results in a positive feedback loop of increased DNA damage and impaired DNA repair [78].

**KLF4** counteracts the suppressive effects of p53 on NHEJ and HR mechanisms to modulate DNA repair [74], and in colitis-associated CRC models, it maintains genomic stability by directing p53 to centrosomes [72]. Meanwhile, the inactivation of the DNA helicase protein **HLTF** in CRC promotes genomic instability and subsequent malignant transformation [81].

### 2.5. ZFPs Modulate p53 Levels

To maintain cell homeostasis through regulation of the cell cycle, DNA repair response, and cell death pathways, p53, a vital tumor suppressor, modulates numerous cell processes via its role as a transcription factor. p53 levels are tightly regulated by numerous proteins, including MDM2, an E3 ubiquitin ligase responsible for p53 degradation, thus maintaining p53 levels appropriate for the cell state [134]. **ZBTB7A**, a tumor suppressor [84], can also function as an oncogene by downregulating p53, thereby enhancing ETS proto-oncogene 1 (ETS-1) function and stimulating CRC cell proliferation and invasion [82]. Both **GLI3** [86] and the p52 isoform of **ZFP398** [88] help stabilize the p53–MDM2 complex by binding to p53 and MDM2. The resulting ubiquitination and exosomal removal of p53 facilitate neoplasia and cell proliferation [88].

### 2.6. ZFPs Modulate E-Cadherin Expression

The transmembrane glycoprotein E-cadherin, a well-studied cell-adhesion molecule, is commonly expressed in epithelial cells to prevent metastatic behavior [135]. In CRC, **SNAI1** upregulation induces EMT and metastasis by inhibiting transcription and subsequent expression of the vitamin D receptor and E-cadherin, and promoting β-catenin nuclear translocation to potentiate Wnt pathway signaling [89]. **SPRY2**, a receptor tyrosine kinase modulator, also downregulates vitamin D_3_-dependent E-cadherin expression to augment cellular invasion and de-differentiation [92]. **NANOS1**, a downstream target gene of E-cadherin, can also compromise the anti-migratory properties of E-cadherin. hNanos1, inversely correlated with E-cadherin expression, induces cytoplasmic translocation of p120 catenin (p120ctn), a regulatory protein that complexes with cadherin proteins at the cell membrane to maintain adherens junctions, subsequently disrupting cell-cell adhesion stability [93]. However, to attenuate cell migration and invasion, **ZC3H12C** overexpression enhances E-cadherin expression and suppresses vimentin expression in human CRC cells without altering cell survival [94].

### 2.7. ZFPs Modulate CRC Cell Metabolism

As opposed to metabolic pathways such as oxidative phosphorylation, CRC cells, like many other cancer cell types, utilize aerobic glycolysis for energy generation. Glutaminolysis, the breakdown of glutamine, is upregulated in CRC to maintain anaplerosis, which sustains glycolytic pathways needed for tumor cell growth and proliferation [136]. **ZBTB7C**, downregulated in CRC, is thought to block Myc and tumor cell glutaminolysis, thus increasing immune cell proliferation due to the glutamine surplus in the tumor microenvironment and attenuating CRC cell proliferation [98]. Enhanced tumor cell aerobic glycolysis is also exploited by several oncogenes to promote cell proliferation and metastasis, e.g., **YY1**, via *GLUT3* transcription upregulation [95], and **ZFP1**, via p53-dependent glycolysis [99]. **ZNF568** can accomplish this by inhibiting p53-mediated mitochondrial metabolism [99].

### 2.8. ZFPs Modulate the Expression of Factors That Stimulate Angiogenesis

A major mechanism underlying CRC metastasis is angiogenesis, the formation of new blood vessels, via modulation of vascular endothelial growth factor (VEGF) signaling [137]. VEGF targets tyrosine kinase receptors to regulate downstream signaling cascades that alter pro-angiogenic behavior such as vascular permeability and cell survival [137]. The availability of VEGF can be upregulated by matrix metalloproteinase-2 (MMP2), a multifunctional protein in cancer [138]. **ZNF384** [100], **SNAI2** [103], and **ZEB2** [107] are upregulated in CRC and enhance MMP2 expression and/or activity, promoting angiogenesis. **SNAI2**, which expedites p53/p21 degradation by upregulating MDM2 [101], is essential for mutant-KRAS cancer cell survival after EMT [104]. **ZNF24** represses VEGF expression [109] to suppress angiogenesis, while **ZKSCAN3**, an inducer of VEGF to promote CRC development and invasion, is implicated in carcinoembryonic antigen (CEA)-producing tumor liver metastasis [110].

## 3. ZFPs Coordinate Cell Cycle Regulation and Apoptotic Mechanisms in CRC (Table 2)

Cell cycle regulation, essential to maintain cell integrity, is monitored at key checkpoints. This process is highly dependent on cyclins, cyclin-dependent kinases (CDKs), and p53, which regulate cell cycle progression and prevent inappropriate cell growth and genomic replication [139]. Apoptosis, induced by cell stress signals, can occur via either the intrinsic or extrinsic pathway; the former is mediated by mitochondrial and Bcl-2 proteins, and the latter is executed by activation of cell death receptors (e.g., TRAIL). Both pathways ultimately activate caspase signaling cascades that promote programmed cell death [140]. Evading cell cycle arrest and apoptosis is essential for CRC cell survival and proliferation and tumor expansion and spread.

**Table 2:** ZFPs modulating cell cycle arrest and apoptosis.

Molecular Mechanism	Gene	Common Alias(es)	Function	Additional Molecular Mechanisms	Cellular Targets	Role in Additional Cell Phenotypes or Behavior	Refs
Checkpoint	*ZFP36L1*	*BRF1*, *Berg36*, *ERF1*, *RNF162B*, *TIS11B*, *cMG1*	TS		p53, cyclin A, cyclin B, cyclin D	Cell proliferation	[141]
	*ZFP36L2*	*BRF2*, *ERF2*, *OOMD13*, *OZEMA13*, *RNF162C*, *TIS11D*					
	*XAF1*	*BIRC4BP*, *HSXIAPAF1*, *XIAPAF1*	TS		Cyclin B, Chk1, Cdc25; XIAP		[142,143]
	*KLF6*	*BCD1*, *CBA1*, *COPEB*, *CPBP*, *GBF*, *PAC1*, *ST12*, *ZF9*	TS		p21, Bax	Cell proliferation	[144]
	*PATZ1*	*ZNF278*, *MAZR*, *RIAZ*, *ZBTB19*, *ZSG*	O	ERK/MAPK pathway	p21, p53, cyclin D1/E1	Cell proliferation	[145]
	*MZF1*	*MZF-1*, *MZF1B*, *ZFP98*, *ZNF42*, *ZSCAN6*	O		p55^PIK^; *Axl*	Migration, invasion	[146,147,148]
Bcl-x_L_ pathway	*ZIC1*	*BAIDCS*, *CRS6*, *ZIC*, *ZNF201*	TS	PI3K/AKT, MAPK pathways	Bcl-x_L_/Bad/Caspase 3 cascade; GADD45B	Cell proliferation	[149]
	*MECOM*	*AML1-EVI-1*, *EVI1*, *MDS1*, *MDS1-EVI1*, *PRDM3*, *RUSAT2*, *MDS1 and EVI1 complex locus*, *KMT8E*	O	TGF-β; TIMP2, DNMT1	BCl-x_L_, ΔNp63	Cell proliferation, invasion, metastasis	[150,151,152,153]
	*CPEB4*	*CPE-BP4*	O		Bcl-xL, Bax	Cell proliferation, invasion	[154]
Epigenetic modifications	*ZBTB33*	*Kaiso*, *ZNF348*	O	cyclin D1/cyclin E1, MTG16	CDKN2A	Cell proliferation	[155,156,157]
	*ZNF304*		O		*p14^ARF^*, *p15^INK4B^*, *p16^INK4A^*		[158]
	*ZC3HAV1*	*ZAP*; *ZC3H2*; *ARTD13*; *PARP13*; *FLB6421*; *ZC3HDC2*	TS		*TRAILR4*		[159]
	*PRDM2*	*HUMHOXY1*, *KMT8*, *MTB-ZF*, *RIZ*, *RIZ1*, *RIZ2*	TS				[160,161]
Other	*PLAGL1*	*LOT1*, *ZAC*, *ZAC1*	TS		PPARγ	Cell differentiation	[162,163]
	*KLF9*	*BTEB*, *BTEB1*	TS		ISG15	Cell proliferation, differentiation	[164]
	*ZFX*	*ZNF926*	O		DUSP5, MAPK signaling	Cell proliferation	[165,166,167]
	*ZNF746*	*PARIS*	O		GSK3β, FWB7, c-Myc	Cell proliferation	[168]
	*RBBP6*	*MY038*, *P2P-R*, *PACT*, *RBQ-1*, *SNAMA*	O		p53		[169]
	*GLI2*	*CJS*, *HPE9*, *PHS2*, *THP1*, *THP2*	O		TGF-β, HIF1-α	Stemness, cell differentiation	[170]
	*GLIS2*	*NKL*, *NPHP7*	O		PUMA	Cell proliferation, migration	[171]
Under investigation	*GFI1*	*SCN2*, *ZNF163*, *GFI1A*	TS			Cell proliferation	[172]
	*ZBTB16*	*PLZF*, *ZNF145*	O			Stemness, cell proliferation	[173]
	*CIZ1*	*LSFR1*, *NP94*, *ZNF356*	O			Cell proliferation	[174,175]

TS = tumor suppressor, O = oncogene; the NCBI Gene database was utilized to determine common alias(es).

### 3.1. ZFPs and Cell Cycle Checkpoint Regulation

The backbone of cell cycle regulation consists of CDKs activated by cyclins in the presence of mitogenic signals [176]. **ZFP36L1** and **ZFP36L2**, both downregulated in CRC, are postulated to promote G1-phase cell cycle arrest without triggering cell death, likely by downregulating cyclin D expression [141]. Other regulatory components, such as checkpoint kinase 1 (Chk1), are vital to ensure cell cycle arrest until DNA damage is repaired [139]. To achieve G2-M cell cycle arrest, **XAF1** activates Chk1 and then inactivates Cdc25C, a CDK activator, and the Cdc2–cyclin B complex [143].

Other promoters of cell cycle arrest following DNA damage include cyclin-dependent kinase inhibitors, such as p21^Cip1/Waf^, which is upregulated by p53 [139]. **KLF6** (particularly the SV2 splice variant implicated in sporadic CRC) induces apoptosis by upregulating p21^Cip1/Waf1^ and Bax, a pro-apoptotic Bcl-2 family protein [144]. Alternatively, **PATZ1**, upregulated in CRC, downregulates p21^Cip1/Waf1^ expression and prevents cell cycle arrest by upregulating cyclin D1 and E1 [145]. **MZF1** induces *p55^PIK^* gene transcription by attaching to the cis-element “TGGGGA”, which in turn promotes cell cycle progression through p55^PIK^–Rb interactions [146]. Interestingly, *MZF1* is also implicated in sulindac sulfide-mediated TRAIL receptor (death receptor 5) transcription, leading to apoptosis—A potential strategy for cancer therapy [147].

### 3.2. Homeostasis Governed by the Bcl-2 Protein Family Can Be Modulated by ZFPs

Bcl2 family proteins, comprising pro- and anti-apoptotic proteins, are differentially regulated to maintain cellular homeostasis. When apoptosis is induced by cell stress, anti-apoptotic proteins (e.g., Bax, Bcl-x_L_) are upregulated by p53-induced BH3-only proteins, which target mitochondrial proteins to initiate caspase activation and programmed cell death [140]. **ZIC1**, downregulated by promoter hypermethylation in CRC, induces apoptosis by triggering the Bcl-x_L_/Bad/Caspase 3 cascade [149]. **MECOM** upregulation in CRC inhibits apoptosis by inducing Bcl-x_L_ protein transcription and augmenting cells in the G0/G1 phase [151]. **CPEB4** promotes CRC development by downregulating *Bax* and increasing *Bcl-*x*_L_* expression, thus suppressing apoptosis [154].

### 3.3. Epigenetic Modifications Modulated by ZFPs

As discussed above, epigenetic modifications provide a common mechanism to regulate ZFP function and can be manipulated by ZFPs to suppress CRC progression. Both **ZBTB33** [157] and **ZNF304** [158] promote tumor suppressor gene silencing by directly binding to CpG island methylator phenotype gene promoters and recruiting corepressor complexes to methylate target genes, thereby preventing CRC cell cycle arrest. In contrast, to induce apoptosis in an exosome-dependent manner, **ZC3HAV1** binds directly to and degrades the mRNA transcript of anti-apoptotic TRAIL decoy receptor 4 *(TRAILR4)* [159]. Interestingly, the tumor-suppressive effects of ZC3HAV1 are dictated by the tumor microenvironment. In the presence of TRAIL signals, ZC3HAV1 stimulates apoptosis, but in their absence, ZC3HAV1 suppresses cell growth. **PRDM2**, frequently inactivated by DNA methylation, has been shown to promote cell apoptosis and G2/M arrest in colon cancer cells [160].

### 3.4. Other Mechanisms Whereby ZFPs Modulate CRC Progression

ZFPs can modulate CRC pathogenesis by modulating cell signaling and dependency on the tumor microenvironment. **PLAGL1**, an anti-proliferative gene, modulates apoptosis and cell cycle arrest in a PPARγ-dependent manner by upregulating the expression of target genes involved in cell growth [162]. **KLF9** inhibits ISG15, an apoptosis-inhibiting cytokine, thereby suppressing tumorigenesis [164], while **ZFX** downregulates DUSP5, permitting constitutive MAPK signaling, to promote apoptotic resistance and cell cycle progression [166].

**ZNF746** upregulation in CRC likely inhibits GSK3β and FWB7 to modulate site-specific phosphorylation and dephosphorylation of oncogenic c-Myc, an action that blocks c-Myc ubiquitination and degradation to ultimately evade G1 cell cycle arrest [168]. **RBBP6** upregulation in CRC facilitates MDM2-mediated p53 ubiquitination and degradation to promote tumorigenesis [169].

**GLI2** activation by HIF-1α and cancer-associated fibroblast-secreted TGF-β2 recruits anti-apoptotic molecules, allowing evasion of chemotherapy-induced apoptosis in a hypoxic tumor microenvironment [170]. To augment cell migration and retard apoptosis, **GLIS2**, a GLI2-related KLF protein, inhibits transcription of the apoptotic gene *PUMA* and focal adhesion genes (e.g., cadherins), likely by modulating acetylation levels of gene enhancers [171].

## 4. ZFPs Aid in Maintaining Cell Stemness to Propagate CRC-Promoting Cell Behaviors (Table 3)

Maintenance of stemness allows the self-renewal, differentiation, and propagation of a specific cell lineage. Cancer cells adopt this phenotype to enhance cell survival, cloning, and proliferation [177]. In CRC, many ZFPs contribute to this stem-like maintenance by manipulating Wnt signaling and epigenetic pathways.

**Table 3:** ZFPs involved in regulating cell stemness.

Molecular Mechanism	Gene	Common Alias(es)	Function	Additional Molecular Mechanisms	Cellular Targets	Role in Additional Cell Phenotypes or Behavior	Refs.
Wnt	*JADE3*	*PHF16*	O		LGR5		[178]
	*PRDM1*	*BLIMP1*, *PRDI-BF1*	O		IGFBP3, ERK1/2		[179]
	*SALL4*	*DRRS*, *HSAL4*, *IVIC*, *ZNF797*	O		β-catenin, GLI2	Metastasis	[180,181,182]
Epigenetics	*ZRANB1*	*Trabid*	O	Wnt signaling, APC	EZH2	Cell proliferation	[183]
	*UPF1*	*HUPF1*, *NORF1*, *RENT1*, *UTF*, *pNORF1*, *smg-2*	O		TOP2A		[184]
	*SALL3*	*ZNF796*	O		Under investigation		[185,186]
Under investigation	PRDM14	*PFM11*	O			Invasion	[187]
	*RBCK1*	*HOIL1*, *PBMEI*, *PGBM1*, *RBCK2*, *RNF54*, *UBCE7IP3*, *XAP3*, *XAP4*, *ZRANB4*	O			Migration and invasion	[188]

O = oncogene; the NCBI Gene database was utilized to determine common alias(es).

### 4.1. ZFPs Modulate Wnt Signaling

To maintain stemness, upregulation of **JADE3** expression in CRC potentiates canonical Wnt signaling and subsequent transcriptional upregulation of the stem cell regulator and marker *LGR5* by recruiting histone acetylases at the *LGR5* promoter [178]. Conversely, **PRDM1** contributes to cancer cell stemness by preferential regulation of the non-canonical Wnt pathway, specifically the planar cell polarity pathway, detected by the presence of upregulated downstream targets such as *Wnt5A* and *Fzd4* [179]. **SALL4** was shown to promote metastasis [180] and stem cell self-renewal via its ability to modulate GLI1 expression [181] and potentiate the canonical Wnt/β-catenin pathway by direct interactions with β-catenin [182].

### 4.2. ZFPs Modulate Epigenetic Modifications

When recruited by lncRNA FAM83C-AS1, **ZRANB1** de-ubiquitinates and stabilizes histone methyltransferase EZH2 to downregulate the expression of the metastasis suppressor *SEMA3F* and increase CRC cell stemness, proliferative, and metastatic capabilities [183]. Interestingly, **UPF1** upregulation induces oxaliplatin chemoresistance by augmenting topoisomerase enzyme activity, thereby sustaining CRC stemness [184].

## 5. ZNF Structure and Function in CRC

In addition to highlighting the vital functions of ZFPs in CRC pathogenesis, it is important to recognize the role ZNF motifs play as core domains enabling ZFPs to execute specific tasks. ZNF motifs house consensus sequences commonly comprising cysteine (Cys) and histidine (His) residues coordinated by one or more zinc ions. The configuration of the ZNF structural fold varies depending on the combination and location of coordinated residues within the consensus sequence. Thirty types of ZNFs are classified based on structural composition, each type likely contributing to specific ZFP functions [189], particularly in CRC progression.

The majority of ZNFs belong to the Cys2His2 (C2H2)-type family. This ZNF family structurally comprises a single α-helix and anti-parallel β-sheet containing pairs of Cys and His residues coordinated by one zinc ion, which is necessary for target DNA sequence recognition and binding [190]. ZNF281, KLF4, and SNAI2 are among several ZFPs that harbor C2H2-type domains and modulate Wnt signaling, DNA damage response mechanisms, and angiogenesis, respectively, to modify CRC cell behavior [189].

E3-ubiquitin ligases, an abundant type of ZFP, can possess RING finger domains, characterized by a consensus sequence containing multiple Cys residues and a single His residue coordinated by two zinc ions. This domain is vital for ZNRF3, which regulates cytoplasmic β-catenin levels to negatively modulate Wnt signaling and cell proliferation [8]. UHRF1 possesses both RING finger and PHD domains responsible for silencing CRC metastatic tumor suppressor genes via epigenetic modifications [189].

### Conserved Mutations in C2H2-Type ZFPs in CRC

The structural importance of ZNF motifs in ZFP function in CRC is evidenced by the functional consequences of ZNF-localized mutations. Computational and functional genomic analyses of CRC tumor samples reveal a high somatic mutation frequency at specific, conserved amino acid residues within C2H2-containing ZFP transcription factors, including ZNF382, ZNF281, and ZEB1 [191]. The most common mutation, an arginine-to-isoleucine missense mutation at position 9, is predicted to alter the necessary orientation of the ZNF with respect to neighboring domains and impair the specificity of target DNA recognition and binding [191]. This, in concert with the finding that adjacent residues (i.e., position 8) are not frequently mutated in CRC [191], highlights the integral role of highly conserved residues in maintaining crucial ZNF structure and ZFP functions. It is possible that there is a threshold for the types of ZNF mutations tolerable for cells, particularly for ZFPs necessary for essential cellular functions. Additional studies are necessary to elucidate the molecular underpinnings by which such mutations modify ZFP-specific functions and downstream target gene expression; this will help lay the groundwork for personalized, targeted therapeutic development.

## 6. Evaluating the Potential of ZFPs as CRC Therapeutic Targets

As these multidimensional proteins play diverse roles in promoting CRC, ZFPs are poised to serve as targets for therapeutic development. A potential approach is the design and use of metallo-compounds to alter zinc coordination properties within the crucial ZNF motif. Several metal-based compounds are approved to treat various conditions, including rheumatoid arthritis, cancer (e.g., cisplatin), and parasitic infestation [192]. In vitro studies targeting SP1 [193] and ZFP36 [194] with copper complexes have demonstrated therapeutic potential by diminishing chemoresistance and modulating inflammation, respectively. Future studies must evaluate the potential side effects of metal-complex treatments and monitor for metal toxicity. Another potential approach to modulate ZFP activity in CRC is to repurpose thalidomide analogs, already approved to treat select hematologic cancers. These small-molecule drugs degrade C2H2-type ZFPs by activating the E3 ubiquitin ligase CRL4^CRBN^ in vitro [195]. The use of thalidomide analogs to target oncogenic C2H2-containing ZFPs involved in promoting CRC may be a promising avenue of exploration.

## 7. Current Limitations to Targeting ZFPs for CRC Therapy

**Figure 2:**
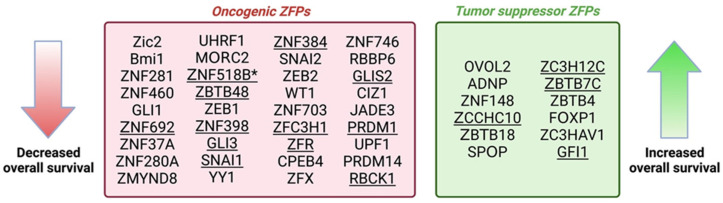
ZFPs implicated in overall survival (OS) outcomes for individuals with colorectal cancer. Increased expression of tumor suppressor ZFPs increased OS, while upregulation of oncogenic ZFPs decreased OS. Underlined ZFPs indicate expression of mRNA transcripts, instead of protein levels. Asterisk (*) indicates a decreased ratio of mRNA isoform 2 to 1. Created with Biorender.com.

While we highlighted specific roles of ZFPs throughout this review, it is important to note that these proteins play multiple roles in the promotion of tumorigenic cell phenotypes (Table 1, Table 2 and Table 3). The ability of ZFPs to cross-regulate multiple different mechanisms, including signaling pathways, cell cycle checkpoints, and angiogenic markers, demonstrates the complexity of investigating the role of distinct ZFPs in CRC pathogenesis and identifying ZFPs as targets with therapeutic potential. While altered expression of certain ZFPs is linked to CRC survival (Figure 2), the precise underlying mechanisms remain elusive for most proteins. Additionally, there are conflicting data regarding whether some ZFPs, e.g., SALL1 [185,196], ZIC5 [197,198], ZNF148 [199,200,201], and ZNF750 [202,203], are CRC tumor suppressors or oncogenes; this raises the possibility they have dual functions depending on tumor stage—additional studies are needed to explore this possibility.

Many studies performed to understand the pathways and signaling mechanisms by which ZFPs suppress or promote CRC behavior stem are limited by the reliance on cell lines or murine models to draw conclusions regarding human disease. Additional work using human tissue, perhaps organoids, will help confirm their putative roles and mechanisms of action. While ZFPs play a complex role in CRC, ZFP lncRNA antisense (AS) transcripts are also emerging as players. ZFAS1 [204], ZEB2-AS1 [205], and ZEB1-AS1 [206] are among the lncRNA transcripts upregulated in CRC. Future studies investigating ZFPs, and their AS transcripts, will expand our understanding of their role in CRC development and progression and the potential to develop novel therapeutics based on this information.

## 8. Conclusions

ZFPs play key roles in CRC development and progression, primarily acting as oncogenes or tumor suppressors, by regulating cell proliferation, senescence, apoptosis, cell cycle, EMT, cell migration, invasion, stemness, and Wnt signaling. Future research can be directed at understanding the interactions between various ZFPs and identifying those with the most important effects on CRC metastasis and survival, thus identifying novel therapeutic opportunities.

## Data Availability

Not applicable.

## References

[1] Sung H., Ferlay J., Siegel R.L., Laversanne M., Soerjomataram I., Jemal A., Bray F. (2021). Global Cancer Statistics 2020: GLOBOCAN Estimates of Incidence and Mortality Worldwide for 36 Cancers in 185 Countries. CA Cancer J. Clin..

[2] Siegel R.L., Wagle N.S., Cercek A., Smith R.A., Jemal A. (2023). Colorectal cancer statistics, 2023. CA Cancer J. Clin..

[3] Morgan E., Arnold M., Gini A., Lorenzoni V., Cabasag C.J., Laversanne M., Vignat J., Ferlay J., Murphy N., Bray F. (2023). Global burden of colorectal cancer in 2020 and 2040: Incidence and mortality estimates from GLOBOCAN. Gut.

[4] Lewandowska A., Rudzki G., Lewandowski T., Stryjkowska-Gora A., Rudzki S. (2022). Risk Factors for the Diagnosis of Colorectal Cancer. Cancer Control.

[5] Pino M.S., Chung D.C. (2010). The chromosomal instability pathway in colon cancer. Gastroenterology.

[6] Laity J.H., Lee B.M., Wright P.E. (2001). Zinc finger proteins: New insights into structural and functional diversity. Curr. Opin. Struct. Biol.

[7] Jen J., Wang Y.C. (2016). Zinc finger proteins in cancer progression. J. Biomed. Sci..

[8] Hao H.X., Xie Y., Zhang Y., Charlat O., Oster E., Avello M., Lei H., Mickanin C., Liu D., Ruffner H. (2012). ZNRF3 promotes Wnt receptor turnover in an R-spondin-sensitive manner. Nature.

[9] Zhao H., Ming T., Tang S., Ren S., Yang H., Liu M., Tao Q., Xu H. (2022). Wnt signaling in colorectal cancer: Pathogenic role and therapeutic target. Mol. Cancer.

[10] Ye G.D., Sun G.B., Jiao P., Chen C., Liu Q.F., Huang X.L., Zhang R., Cai W.Y., Li S.N., Wu J.F. (2016). OVOL2, an Inhibitor of WNT Signaling, Reduces Invasive Activities of Human and Mouse Cancer Cells and Is Down-regulated in Human Colorectal Tumors. Gastroenterology.

[11] Xia L., Gao J., Ma K., Lin H., Chen Y., Luo Q., Lian J. (2021). OVOL2 attenuates the expression of MAP3K8 to suppress epithelial mesenchymal transition in colorectal cancer. Pathol. Res. Pract..

[12] Almozyan S., Coulton J., Babaei-Jadidi R., Nateri A.S. (2021). FLYWCH1, a Multi-Functional Zinc Finger Protein Contributes to the DNA Repair Pathway. Cells.

[13] Muhammad B.A., Almozyan S., Babaei-Jadidi R., Onyido E.K., Saadeddin A., Kashfi S.H., Spencer-Dene B., Ilyas M., Lourdusamy A., Behrens A. (2018). FLYWCH1, a Novel Suppressor of Nuclear beta-Catenin, Regulates Migration and Morphology in Colorectal Cancer. Mol. Cancer Res..

[14] Montorsi L., Guizzetti F., Alecci C., Caporali A., Martello A., Atene C.G., Parenti S., Pizzini S., Zanovello P., Bortoluzzi S. (2016). Loss of ZFP36 expression in colorectal cancer correlates to wnt/ ss-catenin activity and enhances epithelial-to-mesenchymal transition through upregulation of ZEB1, SOX9 and MACC1. Oncotarget.

[15] Wu C., Qiu S., Lu L., Zou J., Li W.F., Wang O., Zhao H., Wang H., Tang J., Chen L. (2014). RSPO2-LGR5 signaling has tumour-suppressive activity in colorectal cancer. Nat. Commun..

[16] Dong X., Liao W., Zhang L., Tu X., Hu J., Chen T., Dai X., Xiong Y., Liang W., Ding C. (2017). RSPO2 suppresses colorectal cancer metastasis by counteracting the Wnt5a/Fzd7-driven noncanonical Wnt pathway. Cancer Lett..

[17] Xu Z., Zheng J., Chen Z., Guo J., Li X., Wang X., Qu C., Yuan L., Cheng C., Sun X. (2021). Multilevel regulation of Wnt signaling by Zic2 in colon cancer due to mutation of beta-catenin. Cell Death Dis..

[18] Huang Y., Wang P., Chen H., Ding Y., Chen Y.G. (2015). Myc-interacting zinc-finger protein 1 positively regulates Wnt signalling by protecting Dishevelled from Dapper1-mediated degradation. Biochem. J..

[19] Wiese K.E., Walz S., von Eyss B., Wolf E., Athineos D., Sansom O., Eilers M. (2013). The role of MIZ-1 in MYC-dependent tumorigenesis. Cold Spring Harb. Perspect. Med..

[20] Du J., Li Y., Li J., Zheng J. (2010). Polycomb group protein Bmi1 expression in colon cancers predicts the survival. Med. Oncol..

[21] Kim J.H., Yoon S.Y., Kim C.N., Joo J.H., Moon S.K., Choe I.S., Choe Y.K., Kim J.W. (2004). The Bmi-1 oncoprotein is overexpressed in human colorectal cancer and correlates with the reduced p16INK4a/p14ARF proteins. Cancer Lett..

[22] Yu F., Zhou C., Zeng H., Liu Y., Li S. (2018). BMI1 activates WNT signaling in colon cancer by negatively regulating the WNT antagonist IDAX. Biochem. Biophys. Res. Commun..

[23] Zhang Z., Bu X., Chen H., Wang Q., Sha W. (2016). Bmi-1 promotes the invasion and migration of colon cancer stem cells through the downregulation of E-cadherin. Int. J. Mol. Med..

[24] Li N., Li D., Du Y., Su C., Yang C., Lin C., Li X., Hu G. (2019). Overexpressed PLAGL2 transcriptionally activates Wnt6 and promotes cancer development in colorectal cancer. Oncol. Rep..

[25] Lv Y., Xie B., Bai B., Shan L., Zheng W., Huang X., Zhu H. (2019). Weighted gene coexpression analysis indicates that PLAGL2 and POFUT1 are related to the differential features of proximal and distal colorectal cancer. Oncol. Rep..

[26] Strubberg A.M., Veronese Paniagua D.A., Zhao T., Dublin L., Pritchard T., Bayguinov P.O., Fitzpatrick J.A.J., Madison B.B. (2018). The Zinc Finger Transcription Factor PLAGL2 Enhances Stem Cell Fate and Activates Expression of ASCL2 in Intestinal Epithelial Cells. Stem Cell Rep..

[27] Qin C.J., Bu P.L., Zhang Q., Chen J.T., Li Q.Y., Liu J.T., Dong H.C., Ren X.Q. (2019). ZNF281 Regulates Cell Proliferation, Migration and Invasion in Colorectal Cancer through Wnt/beta-Catenin Signaling. Cell Physiol. Biochem..

[28] Belaguli N.S., Aftab M., Rigi M., Zhang M., Albo D., Berger D.H. (2010). GATA6 promotes colon cancer cell invasion by regulating urokinase plasminogen activator gene expression. Neoplasia.

[29] Zhao P., Guan H.T., Dai Z.J., Ma Y.G., Liu X.X., Wang X.J. (2017). Knockdown of Tripartite Motif-Containing Protein 37 (TRIM37) Inhibits the Proliferation and Tumorigenesis in Colorectal Cancer Cells. Oncol. Res..

[30] Cheng K., Xie G., Khurana S., Heath J., Drachenberg C.B., Timmons J., Shah N., Raufman J.P. (2014). Divergent effects of muscarinic receptor subtype gene ablation on murine colon tumorigenesis reveals association of M3R and zinc finger protein 277 expression in colon neoplasia. Mol. Cancer.

[31] Xie G., Peng Z., Liang J., Larabee S.M., Drachenberg C.B., Yfantis H., Raufman J.P. (2022). Zinc finger protein 277 is an intestinal transit-amplifying cell marker and colon cancer oncogene. JCI Insight.

[32] Blaj C., Bringmann A., Schmidt E.M., Urbischek M., Lamprecht S., Frohlich T., Arnold G.J., Krebs S., Blum H., Hermeking H. (2017). ADNP Is a Therapeutically Inducible Repressor of WNT Signaling in Colorectal Cancer. Clin. Cancer Res..

[33] Zhu D., Zhou J., Zhao J., Jiang G., Zhang X., Zhang Y., Dong M. (2019). ZC3H13 suppresses colorectal cancer proliferation and invasion via inactivating Ras-ERK signaling. J. Cell Physiol..

[34] Mansilla F., Birkenkamp-Demtroder K., Kruhoffer M., Sorensen F.B., Andersen C.L., Laiho P., Aaltonen L.A., Verspaget H.W., Orntoft T.F. (2007). Differential expression of DHHC9 in microsatellite stable and instable human colorectal cancer subgroups. Br. J. Cancer.

[35] Verma V., Paek A.R., Choi B.K., Hong E.K., You H.J. (2019). Loss of zinc-finger protein 143 contributes to tumour progression by interleukin-8-CXCR axis in colon cancer. J. Cell. Mol. Med..

[36] Paek A.R., Lee C.H., You H.J. (2014). A role of zinc-finger protein 143 for cancer cell migration and invasion through ZEB1 and E-cadherin in colon cancer cells. Mol. Carcinog..

[37] Hao T., Xu J., Fang S., Jiang J., Chen X., Wu W., Li L., Li M., Zhang C., He Y. (2021). Overexpression of ZNF460 predicts worse survival and promotes metastasis through JAK2/STAT3 signaling pathway in patient with colon cancer. J. Cancer.

[38] Ma Z.H., Shi P.D., Wan B.S. (2021). MiR-410-3p activates the NF-kappaB pathway by targeting ZCCHC10 to promote migration, invasion and EMT of colorectal cancer. Cytokine.

[39] Ma J., Mi C., Wang K.S., Lee J.J., Jin X. (2016). Zinc finger protein 91 (ZFP91) activates HIF-1alpha via NF-kappaB/p65 to promote proliferation and tumorigenesis of colon cancer. Oncotarget.

[40] Zhao Z., Qin X. (2020). MicroRNA-708 targeting ZNF549 regulates colon adenocarcinoma development through PI3K/AKt pathway. Sci. Rep..

[41] Yang Z., Zhang C., Qi W., Cui Y., Xuan Y. (2018). GLI1 promotes cancer stemness through intracellular signaling pathway PI3K/Akt/NFkappaB in colorectal adenocarcinoma. Exp. Cell Res..

[42] Zhang C., Wang Y., Feng Y., Zhang Y., Ji B., Wang S., Sun Y., Zhu C., Zhang D., Sun Y. (2016). Gli1 promotes colorectal cancer metastasis in a Foxm1-dependent manner by activating EMT and PI3K-AKT signaling. Oncotarget.

[43] Xing Y., Ren S., Ai L., Sun W., Zhao Z., Jiang F., Zhu Y., Piao D. (2019). ZNF692 promotes colon adenocarcinoma cell growth and metastasis by activating the PI3K/AKT pathway. Int. J. Oncol..

[44] Akhtar Ali M., Younis S., Wallerman O., Gupta R., Andersson L., Sjoblom T. (2015). Transcriptional modulator ZBED6 affects cell cycle and growth of human colorectal cancer cells. Proc. Natl. Acad. Sci. USA.

[45] Liu J., Huang Z., Chen H.N., Qin S., Chen Y., Jiang J., Zhang Z., Luo M., Ye Q., Xie N. (2021). ZNF37A promotes tumor metastasis through transcriptional control of THSD4/TGF-beta axis in colorectal cancer. Oncogene.

[46] Yang Y., Yan T., Han Q., Zhang M., Zhang Y., Luo Y., Wei L., Li P., Wang E. (2021). ZNF326 promotes colorectal cancer epithelial-mesenchymal transition. Pathol. Res. Pract..

[47] Lei T., Gao Y., Duan Y., Cui C., Zhang L., Si M. (2021). Inhibition of zinc finger protein 367 exerts a tumor suppressive role in colorectal cancer by affecting the activation of oncogenic YAP1 signaling. Environ. Toxicol..

[48] Wang X., Sun D., Tai J., Chen S., Hong S., Wang L. (2019). ZNF280A Promotes Proliferation and Tumorigenicity via Inactivating the Hippo-Signaling Pathway in Colorectal Cancer. Mol. Ther. Oncolytics.

[49] Chen J., He Q., Wu P., Fu J., Xiao Y., Chen K., Xie D., Zhang X. (2020). ZMYND8 expression combined with pN and pM classification as a novel prognostic prediction model for colorectal cancer: Based on TCGA and GEO database analysis. Cancer Biomark..

[50] Pan Q., Zhong S., Wang H., Wang X., Li N., Li Y., Zhang G., Yuan H., Lian Y., Chen Q. (2021). The ZMYND8-regulated mevalonate pathway endows YAP-high intestinal cancer with metabolic vulnerability. Mol. Cell.

[51] Bazzocco S., Dopeso H., Martinez-Barriocanal A., Anguita E., Nieto R., Li J., Garcia-Vidal E., Maggio V., Rodrigues P., de Marcondes P.G. (2021). Identification of ZBTB18 as a novel colorectal tumor suppressor gene through genome-wide promoter hypermethylation analysis. Clin. Epigenetics.

[52] Hellebrekers D.M., Lentjes M.H., van den Bosch S.M., Melotte V., Wouters K.A., Daenen K.L., Smits K.M., Akiyama Y., Yuasa Y., Sanduleanu S. (2009). GATA4 and GATA5 are potential tumor suppressors and biomarkers in colorectal cancer. Clin. Cancer Res..

[53] Siraj A.K., Parvathareddy S.K., Siraj N., Al-Obaisi K., Aldughaither S.M., AlManea H.M., AlHussaini H.F., Al-Dayel F., Al-Kuraya K.S. (2021). Loss of ZNF677 expression is a predictive biomarker for lymph node metastasis in Middle Eastern Colorectal Cancer. Sci. Rep..

[54] Wang S., Wong C.C., Zhang Y., Huang J., Li C., Zhai J., Wang G., Wei H., Zhang X., He H.H. (2021). ZNF545 loss promotes ribosome biogenesis and protein translation to initiate colorectal tumorigenesis in mice. Oncogene.

[55] Zhang S., Xiao J., Chai Y., Hong Z., Liu Z., Yuan R., Luo Z., Zhou X., Lucero-Prisno D.E., Huang K. (2018). Speckle-Type POZ Protein Down-Regulates Matrix Metalloproteinase 2 Expression via Sp1/PI3K/Akt Signaling Pathway in Colorectal Cancer. Dig. Dis. Sci..

[56] Song Y., Xu Y., Pan C., Yan L., Wang Z.W., Zhu X. (2020). The emerging role of SPOP protein in tumorigenesis and cancer therapy. Mol. Cancer.

[57] Xu J., Wang F., Jiang H., Jiang Y., Chen J., Qin J. (2015). Properties and Clinical Relevance of Speckle-Type POZ Protein in Human Colorectal Cancer. J. Gastrointest. Surg..

[58] Watanabe Y., Toyota M., Kondo Y., Suzuki H., Imai T., Ohe-Toyota M., Maruyama R., Nojima M., Sasaki Y., Sekido Y. (2007). PRDM5 identified as a target of epigenetic silencing in colorectal and gastric cancer. Clin. Cancer Res..

[59] Kong X., Chen J., Xie W., Brown S.M., Cai Y., Wu K., Fan D., Nie Y., Yegnasubramanian S., Tiedemann R.L. (2019). Defining UHRF1 Domains that Support Maintenance of Human Colon Cancer DNA Methylation and Oncogenic Properties. Cancer Cell.

[60] Du B., Yang Z.Y., Zhong X.Y., Fang M., Yan Y.R., Qi G.L., Pan Y.L., Zhou X.L. (2011). Metastasis-associated protein 1 induces VEGF-C and facilitates lymphangiogenesis in colorectal cancer. World J. Gastroenterol..

[61] Malisetty V.L., Penugurti V., Panta P., Chitta S.K., Manavathi B. (2017). MTA1 expression in human cancers—Clinical and pharmacological significance. Biomed. Pharmacother..

[62] Liu J., Shao Y., He Y., Ning K., Cui X., Liu F., Wang Z., Li F. (2019). MORC2 promotes development of an aggressive colorectal cancer phenotype through inhibition of NDRG1. Cancer Sci..

[63] Gimeno-Valiente F., Riffo-Campos A.L., Torres L., Tarazona N., Gambardella V., Cervantes A., Lopez-Rodas G., Franco L., Castillo J. (2021). Epigenetic Mechanisms Are Involved in the Oncogenic Properties of ZNF518B in Colorectal Cancer. Cancers.

[64] Antoine K., Ferbus D., Kolahgar G., Prosperi M.T., Goubin G. (2005). Zinc finger protein overexpressed in colon carcinoma interacts with the telomeric protein hRap1. J. Cell. Biochem..

[65] Zhu S., Chen C.Y., Hao Y. (2021). LncRNA KCNQ1OT1 acts as miR-216b-5p sponge to promote colorectal cancer progression via up-regulating ZNF146. J. Mol. Histol..

[66] Cheng Y., Geng H., Cheng S.H., Liang P., Bai Y., Li J., Srivastava G., Ng M.H., Fukagawa T., Wu X. (2010). KRAB zinc finger protein ZNF382 is a proapoptotic tumor suppressor that represses multiple oncogenes and is commonly silenced in multiple carcinomas. Cancer Res..

[67] Liao Q., Chen L., Zhang N., Xi Y., Hu S., Ng D.M., Ahmed F.Y.H., Zhao G., Fan X., Xie Y. (2020). Network analysis of KLF5 targets showing the potential oncogenic role of SNHG12 in colorectal cancer. Cancer Cell Int..

[68] Bajpai R., Nagaraju G.P. (2017). Specificity protein 1: Its role in colorectal cancer progression and metastasis. Crit. Rev. Oncol. Hematol..

[69] Chen X., Zeng K., Xu M., Hu X., Liu X., Xu T., He B., Pan Y., Sun H., Wang S. (2018). SP1-induced lncRNA-ZFAS1 contributes to colorectal cancer progression via the miR-150-5p/VEGFA axis. Cell Death Dis..

[70] Parker J.B., Palchaudhuri S., Yin H., Wei J., Chakravarti D. (2012). A transcriptional regulatory role of the THAP11-HCF-1 complex in colon cancer cell function. Mol. Cell. Biol..

[71] Jung S.J., Seo Y.R., Park W.J., Heo Y.R., Lee Y.H., Kim S., Lee J.H. (2020). Clinicopathological Characteristics of TZAP Expression in Colorectal Cancers. Onco Targets Ther..

[72] Yang V.W., Liu Y., Kim J., Shroyer K.R., Bialkowska A.B. (2019). Increased Genetic Instability and Accelerated Progression of Colitis-Associated Colorectal Cancer through Intestinal Epithelium-specific Deletion of Klf4. Mol. Cancer Res..

[73] Ma Y., Wu L., Liu X., Xu Y., Shi W., Liang Y., Yao L., Zheng J., Zhang J. (2017). KLF4 inhibits colorectal cancer cell proliferation dependent on NDRG2 signaling. Oncol. Rep..

[74] Ghaleb A.M., Elkarim E.A., Bialkowska A.B., Yang V.W. (2016). KLF4 Suppresses Tumor Formation in Genetic and Pharmacological Mouse Models of Colonic Tumorigenesis. Mol. Cancer Res..

[75] Wang H., Yang L., Jamaluddin M.S., Boyd D.D. (2004). The Kruppel-like KLF4 transcription factor, a novel regulator of urokinase receptor expression, drives synthesis of this binding site in colonic crypt luminal surface epithelial cells. J. Biol. Chem..

[76] Wang F., Sun G., Peng C., Chen J., Quan J., Wu C., Lian X., Tang W., Xiang D. (2021). ZEB1 promotes colorectal cancer cell invasion and disease progression by enhanced LOXL2 transcription. Int. J. Clin. Exp. Pathol..

[77] Sanchez-Tillo E., de Barrios O., Siles L., Amendola P.G., Darling D.S., Cuatrecasas M., Castells A., Postigo A. (2013). ZEB1 Promotes invasiveness of colorectal carcinoma cells through the opposing regulation of uPA and PAI-1. Clin. Cancer Res..

[78] de Barrios O., Sanchez-Moral L., Cortes M., Ninfali C., Profitos-Peleja N., Martinez-Campanario M.C., Siles L., Del Campo R., Fernandez-Acenero M.J., Darling D.S. (2019). ZEB1 promotes inflammation and progression towards inflammation-driven carcinoma through repression of the DNA repair glycosylase MPG in epithelial cells. Gut.

[79] Wu D.W., Lin P.L., Cheng Y.W., Huang C.C., Wang L., Lee H. (2016). DDX3 enhances oncogenic KRAS-induced tumor invasion in colorectal cancer via the beta-catenin/ZEB1 axis. Oncotarget.

[80] Li Y., Wang L., Pappan L., Galliher-Beckley A., Shi J. (2012). IL-1beta promotes stemness and invasiveness of colon cancer cells through Zeb1 activation. Mol. Cancer.

[81] Sandhu S., Wu X., Nabi Z., Rastegar M., Kung S., Mai S., Ding H. (2012). Loss of HLTF function promotes intestinal carcinogenesis. Mol. Cancer.

[82] Zhu M., Li M., Zhang F., Feng F., Chen W., Yang Y., Cui J., Zhang D., Linghu E. (2014). FBI-1 enhances ETS-1 signaling activity and promotes proliferation of human colorectal carcinoma cells. PLoS ONE.

[83] Wang Z., Zhao X., Wang W., Liu Y., Li Y., Gao J., Wang C., Zhou M., Liu R., Xu G. (2018). ZBTB7 evokes 5-fluorouracil resistance in colorectal cancer through the NFkappaB signaling pathway. Int. J. Oncol..

[84] Liu X.S., Liu Z., Gerarduzzi C., Choi D.E., Ganapathy S., Pandolfi P.P., Yuan Z.M. (2016). Somatic human ZBTB7A zinc finger mutations promote cancer progression. Oncogene.

[85] Singh A.K., Verma S., Kushwaha P.P., Prajapati K.S., Shuaib M., Kumar S., Gupta S. (2021). Role of ZBTB7A zinc finger in tumorigenesis and metastasis. Mol. Biol. Rep..

[86] Kang H.N., Oh S.C., Kim J.S., Yoo Y.A. (2012). Abrogation of Gli3 expression suppresses the growth of colon cancer cells via activation of p53. Exp. Cell Res..

[87] Shen M., Zhang Z., Wang P. (2021). GLI3 Promotes Invasion and Predicts Poor Prognosis in Colorectal Cancer. BioMed Res. Int..

[88] Huang C., Wu S., Li W., Herkilini A., Miyagishi M., Zhao H., Kasim V. (2019). Zinc-finger protein p52-ZER6 accelerates colorectal cancer cell proliferation and tumour progression through promoting p53 ubiquitination. EBioMedicine.

[89] Brzozowa M., Michalski M., Wyrobiec G., Piecuch A., Dittfeld A., Harabin-Slowinska M., Boron D., Wojnicz R. (2015). The role of Snail1 transcription factor in colorectal cancer progression and metastasis. Contemp. Oncol..

[90] Beyes S., Andrieux G., Schrempp M., Aicher D., Wenzel J., Anton-Garcia P., Boerries M., Hecht A. (2019). Genome-wide mapping of DNA-binding sites identifies stemness-related genes as directly repressed targets of SNAIL1 in colorectal cancer cells. Oncogene.

[91] Mohammadpour S., Torshizi Esfahani A., Karimpour R., Bakhshian F., Mortazavi Tabatabaei S.A., Laleh A., Nazemalhosseini-Mojarad E. (2019). High expression of Snail1 is associated with EMAST and poor prognosis in CRC patients. Gastroenterol. Hepatol. Bed Bench.

[92] Barbachano A., Ordonez-Moran P., Garcia J.M., Sanchez A., Pereira F., Larriba M.J., Martinez N., Hernandez J., Landolfi S., Bonilla F. (2010). SPROUTY-2 and E-cadherin regulate reciprocally and dictate colon cancer cell tumourigenicity. Oncogene.

[93] Strumane K., Bonnomet A., Stove C., Vandenbroucke R., Nawrocki-Raby B., Bruyneel E., Mareel M., Birembaut P., Berx G., van Roy F. (2006). E-cadherin regulates human Nanos1, which interacts with p120ctn and induces tumor cell migration and invasion. Cancer Res..

[94] Suk F.M., Chang C.C., Lin R.J., Lin S.Y., Chen Y.T., Liang Y.C. (2018). MCPIP3 as a Potential Metastasis Suppressor Gene in Human Colorectal Cancer. Int. J. Mol. Sci..

[95] Wang Y., Wu S., Huang C., Li Y., Zhao H., Kasim V. (2018). Yin Yang 1 promotes the Warburg effect and tumorigenesis via glucose transporter GLUT3. Cancer Sci..

[96] Zhang N., Li X., Wu C.W., Dong Y., Cai M., Mok M.T., Wang H., Chen J., Ng S.S., Chen M. (2013). microRNA-7 is a novel inhibitor of YY1 contributing to colorectal tumorigenesis. Oncogene.

[97] Chen X., Jiang Z., Pu Y., Jiang X., Xiang L., Jiang Z. (2020). Zinc finger and BTB domain-containing 7C (ZBTB7C) expression as an independent prognostic factor for colorectal cancer and its relevant molecular mechanisms. Am. J. Transl. Res..

[98] Chen X., Jiang Z., Wang Z., Jiang Z. (2021). The prognostic and immunological effects of ZBTB7C across cancers: Friend or foe?. Aging.

[99] Wang S., Peng Z., Wang S., Yang L., Chen Y., Kong X., Song S., Pei P., Tian C., Yan H. (2018). KRAB-type zinc-finger proteins PITA and PISA specifically regulate p53-dependent glycolysis and mitochondrial respiration. Cell Res..

[100] Yan Z., Zhou Y., Yang Y., Zeng C., Li P., Tian H., Tang X., Zhang G. (2022). Zinc finger protein 384 enhances colorectal cancer metastasis by upregulating MMP2. Oncol. Rep..

[101] Kim J., Lee J., Kim U., Park J.K., Um H.D. (2021). Slug promotes p53 and p21 protein degradation by inducing Mdm2 expression in HCT116 colon cancer cells. Oncol. Lett..

[102] Shioiri M., Shida T., Koda K., Oda K., Seike K., Nishimura M., Takano S., Miyazaki M. (2006). Slug expression is an independent prognostic parameter for poor survival in colorectal carcinoma patients. Br. J. Cancer.

[103] Welch-Reardon K.M., Ehsan S.M., Wang K., Wu N., Newman A.C., Romero-Lopez M., Fong A.H., George S.C., Edwards R.A., Hughes C.C. (2014). Angiogenic sprouting is regulated by endothelial cell expression of Slug. J. Cell Sci..

[104] Wang Y., Ngo V.N., Marani M., Yang Y., Wright G., Staudt L.M., Downward J. (2010). Critical role for transcriptional repressor Snail2 in transformation by oncogenic RAS in colorectal carcinoma cells. Oncogene.

[105] Geng L., Chaudhuri A., Talmon G., Wisecarver J.L., Are C., Brattain M., Wang J. (2014). MicroRNA-192 suppresses liver metastasis of colon cancer. Oncogene.

[106] Kahlert C., Lahes S., Radhakrishnan P., Dutta S., Mogler C., Herpel E., Brand K., Steinert G., Schneider M., Mollenhauer M. (2011). Overexpression of ZEB2 at the invasion front of colorectal cancer is an independent prognostic marker and regulates tumor invasion in vitro. Clin. Cancer Res..

[107] Li M.Z., Wang J.J., Yang S.B., Li W.F., Xiao L.B., He Y.L., Song X.M. (2017). ZEB2 promotes tumor metastasis and correlates with poor prognosis of human colorectal cancer. Am. J. Transl. Res..

[108] Sreekumar R., Harris S., Moutasim K., DeMateos R., Patel A., Emo K., White S., Yagci T., Tulchinsky E., Thomas G. (2018). Assessment of Nuclear ZEB2 as a Biomarker for Colorectal Cancer Outcome and TNM Risk Stratification. JAMA Netw. Open.

[109] Harper J., Yan L., Loureiro R.M., Wu I., Fang J., D’Amore P.A., Moses M.A. (2007). Repression of vascular endothelial growth factor expression by the zinc finger transcription factor ZNF24. Cancer Res..

[110] Kim C.W., Roh S.A., Tak K.H., Koh B.M., Ha Y.J., Cho D.H., Kim S.Y., Kim Y.S., Kim J.C. (2016). ZKSCAN3 Facilitates Liver Metastasis of Colorectal Cancer Associated with CEA-expressing Tumor. Anticancer Res..

[111] Yang L., Hamilton S.R., Sood A., Kuwai T., Ellis L., Sanguino A., Lopez-Berestein G., Boyd D.D. (2008). The previously undescribed ZKSCAN3 (ZNF306) is a novel “driver” of colorectal cancer progression. Cancer Res..

[112] Bejrananda T., Phukaoloun M., Boonpipattanapong T., Wanitsuwan W., Kanngern S., Sangthong R., Sangkhathat S. (2010). WT1 expression as an independent marker of poor prognosis in colorectal cancers. Cancer Biomark..

[113] Oji Y., Yamamoto H., Nomura M., Nakano Y., Ikeba A., Nakatsuka S., Abeno S., Kiyotoh E., Jomgeow T., Sekimoto M. (2003). Overexpression of the Wilms’ tumor gene WT1 in colorectal adenocarcinoma. Cancer Sci..

[114] Xiang T., He K., Wang S., Chen W., Li H. (2020). Expression of Zinc Finger and BTB Domain-Containing 4 in Colorectal Cancer and Its Clinical Significance. Cancer Manag. Res..

[115] Banham A.H., Beasley N., Campo E., Fernandez P.L., Fidler C., Gatter K., Jones M., Mason D.Y., Prime J.E., Trougouboff P. (2001). The FOXP1 winged helix transcription factor is a novel candidate tumor suppressor gene on chromosome 3p. Cancer Res..

[116] De Smedt L., Palmans S., Govaere O., Moisse M., Boeckx B., De Hertogh G., Prenen H., Van Cutsem E., Tejpar S., Tousseyn T. (2015). Expression of FOXP1 and Colorectal Cancer Prognosis. Lab. Med..

[117] Furukawa D., Chijiwa T., Matsuyama M., Mukai M., Matsuo E.I., Nishimura O., Kawai K., Suemizu H., Hiraoka N., Nakagohri T. (2014). Zinc finger protein 185 is a liver metastasis-associated factor in colon cancer patients. Mol. Clin. Oncol..

[118] Zhang Z.C., Zheng L.Q., Pan L.J., Guo J.X., Yang G.S. (2015). ZNF217 is overexpressed and enhances cell migration and invasion in colorectal carcinoma. Asian Pac. J. Cancer Prev..

[119] Ma F., Bi L., Yang G., Zhang M., Liu C., Zhao Y., Wang Y., Wang J., Bai Y., Zhang Y. (2014). ZNF703 promotes tumor cell proliferation and invasion and predicts poor prognosis in patients with colorectal cancer. Oncol. Rep..

[120] He G., Zou L., Zhou L., Gao P., Qian X., Cui J. (2017). Cysteine-Rich Intestinal Protein 1 Silencing Inhibits Migration and Invasion in Human Colorectal Cancer. Cell. Physiol. Biochem..

[121] He G., Zhu H., Yao Y., Chai H., Wang Y., Zhao W., Fu S., Wang Y. (2019). Cysteine-rich intestinal protein 1 silencing alleviates the migration and invasive capability enhancement induced by excessive zinc supplementation in colorectal cancer cells. Am. J. Transl. Res..

[122] Liang W., Chen W., Wei J., Yao H., Shi J., Hou X., Deng Y., Ou M. (2022). Zinc finger C3H1-type containing serves as a novel prognostic biomarker in human pan-cancer. Gene.

[123] Long Y., Marian T.A., Wei Z. (2019). ZFR promotes cell proliferation and tumor development in colorectal and liver cancers. Biochem. Biophys. Res. Commun..

[124] Hallen L., Klein H., Stoschek C., Wehrmeyer S., Nonhoff U., Ralser M., Wilde J., Rohr C., Schweiger M.R., Zatloukal K. (2011). The KRAB-containing zinc-finger transcriptional regulator ZBRK1 activates SCA2 gene transcription through direct interaction with its gene product, ataxin-2. Hum. Mol. Genet..

[125] Gujral T.S., Chan M., Peshkin L., Sorger P.K., Kirschner M.W., MacBeath G. (2014). A noncanonical Frizzled2 pathway regulates epithelial-mesenchymal transition and metastasis. Cell.

[126] Gargalionis A.N., Papavassiliou K.A., Papavassiliou A.G. (2021). Targeting STAT3 Signaling Pathway in Colorectal Cancer. Biomedicines.

[127] Soleimani A., Rahmani F., Ferns G.A., Ryzhikov M., Avan A., Hassanian S.M. (2020). Role of the NF-kappaB signaling pathway in the pathogenesis of colorectal cancer. Gene.

[128] Danielsen S.A., Eide P.W., Nesbakken A., Guren T., Leithe E., Lothe R.A. (2015). Portrait of the PI3K/AKT pathway in colorectal cancer. Biochim. Biophys. Acta.

[129] Xu Y., Pasche B. (2007). TGF-beta signaling alterations and susceptibility to colorectal cancer. Hum. Mol. Genet..

[130] Lao V.V., Grady W.M. (2011). Epigenetics and colorectal cancer. Nat. Rev. Gastroenterol. Hepatol..

[131] Jia Y., Guo M. (2013). Epigenetic changes in colorectal cancer. Chin. J. Cancer.

[132] Groelly F.J., Fawkes M., Dagg R.A., Blackford A.N., Tarsounas M. (2023). Targeting DNA damage response pathways in cancer. Nat. Rev. Cancer.

[133] Alhmoud J.F., Woolley J.F., Al Moustafa A.E., Malki M.I. (2020). DNA Damage/Repair Management in Cancers. Cancers.

[134] Liebl M.C., Hofmann T.G. (2021). The Role of p53 Signaling in Colorectal Cancer. Cancers.

[135] Paschos K.A., Canovas D., Bird N.C. (2009). The role of cell adhesion molecules in the progression of colorectal cancer and the development of liver metastasis. Cell. Signal..

[136] Brown R.E., Short S.P., Williams C.S. (2018). Colorectal Cancer and Metabolism. Curr. Colorectal. Cancer Rep..

[137] Mousa L., Salem M.E., Mikhail S. (2015). Biomarkers of Angiogenesis in Colorectal Cancer. Biomark. Cancer.

[138] Egeblad M., Werb Z. (2002). New functions for the matrix metalloproteinases in cancer progression. Nat. Rev. Cancer.

[139] Barnum K.J., O’Connell M.J. (2014). Cell cycle regulation by checkpoints. Methods Mol. Biol..

[140] Zhang L., Yu J. (2013). Role of apoptosis in colon cancer biology, therapy, and prevention. Curr. Colorectal. Cancer Rep..

[141] Suk F.M., Chang C.C., Lin R.J., Lin S.Y., Liu S.C., Jau C.F., Liang Y.C. (2018). ZFP36L1 and ZFP36L2 inhibit cell proliferation in a cyclin D-dependent and p53-independent manner. Sci. Rep..

[142] Liston P., Fong W.G., Kelly N.L., Toji S., Miyazaki T., Conte D., Tamai K., Craig C.G., McBurney M.W., Korneluk R.G. (2001). Identification of XAF1 as an antagonist of XIAP anti-Caspase activity. Nat. Cell Biol..

[143] Wang J., Gu Q., Li M., Zhang W., Yang M., Zou B., Chan S., Qiao L., Jiang B., Tu S. (2009). Identification of XAF1 as a novel cell cycle regulator through modulating G(2)/M checkpoint and interaction with checkpoint kinase 1 in gastrointestinal cancer. Carcinogenesis.

[144] Zhang B., Guo D.D., Zheng J.Y., Wu Y.A. (2018). Expression of KLF6-SV2 in colorectal cancer and its impact on proliferation and apoptosis. Eur. J. Cancer Prev..

[145] Tian X.Q., Guo F.F., Sun D.F., Wang Y.C., Yang L., Chen S.L., Hong J., Fang J.Y. (2017). Downregulation of ZNF278 arrests the cell cycle and decreases the proliferation of colorectal cancer cells via inhibition of the ERK/MAPK pathway. Oncol. Rep..

[146] Deng Y., Wang J., Wang G., Jin Y., Luo X., Xia X., Gong J., Hu J. (2013). p55PIK transcriptionally activated by MZF1 promotes colorectal cancer cell proliferation. BioMed Res. Int..

[147] Horinaka M., Yoshida T., Tomosugi M., Yasuda S., Sowa Y., Sakai T. (2014). Myeloid zinc finger 1 mediates sulindac sulfide-induced upregulation of death receptor 5 of human colon cancer cells. Sci. Rep..

[148] Mudduluru G., Vajkoczy P., Allgayer H. (2010). Myeloid zinc finger 1 induces migration, invasion, and in vivo metastasis through Axl gene expression in solid cancer. Mol. Cancer Res..

[149] Gan L., Chen S., Zhong J., Wang X., Lam E.K., Liu X., Zhang J., Zhou T., Yu J., Si J. (2011). ZIC1 is downregulated through promoter hypermethylation, and functions as a tumor suppressor gene in colorectal cancer. PLoS ONE.

[150] Pradeepa, Suresh V., Singh V.K., Nayak K.B., Senapati S., Chakraborty S. (2022). EVI1 promotes metastasis by downregulating TIMP2 in metastatic colon and breast cancer cells. Int. J. Biochem. Cell Biol..

[151] Liang B., Wang J. (2020). EVI1 in Leukemia and Solid Tumors. Cancers.

[152] Nayak K.B., Kuila N., Das Mohapatra A., Panda A.K., Chakraborty S. (2013). EVI1 targets DeltaNp63 and upregulates the cyclin dependent kinase inhibitor p21 independent of p53 to delay cell cycle progression and cell proliferation in colon cancer cells. Int. J. Biochem. Cell Biol..

[153] Deng X., Cao Y., Liu Y., Li F., Sambandam K., Rajaraman S., Perkins A.S., Fields A.P., Hellmich M.R., Townsend C.M. (2013). Overexpression of Evi-1 oncoprotein represses TGF-beta signaling in colorectal cancer. Mol. Carcinog..

[154] Zhong X., Xiao Y., Chen C., Wei X., Hu C., Ling X., Liu X. (2015). MicroRNA-203-mediated posttranscriptional deregulation of CPEB4 contributes to colorectal cancer progression. Biochem. Biophys. Res. Commun..

[155] Barrett C.W., Smith J.J., Lu L.C., Markham N., Stengel K.R., Short S.P., Zhang B., Hunt A.A., Fingleton B.M., Carnahan R.H. (2012). Kaiso directs the transcriptional corepressor MTG16 to the Kaiso binding site in target promoters. PLoS ONE.

[156] Pozner A., Terooatea T.W., Buck-Koehntop B.A. (2016). Cell-specific Kaiso (ZBTB33) Regulation of Cell Cycle through Cyclin D1 and Cyclin E1. J. Biol. Chem..

[157] Lopes E.C., Valls E., Figueroa M.E., Mazur A., Meng F.G., Chiosis G., Laird P.W., Schreiber-Agus N., Greally J.M., Prokhortchouk E. (2008). Kaiso contributes to DNA methylation-dependent silencing of tumor suppressor genes in colon cancer cell lines. Cancer Res..

[158] Serra R.W., Fang M., Park S.M., Hutchinson L., Green M.R. (2014). A KRAS-directed transcriptional silencing pathway that mediates the CpG island methylator phenotype. Elife.

[159] Cai J., Liu W., Wong C.W., Zhu W., Lin Y., Hu J., Xu W., Zhang J., Sander M., Wang Z. (2020). Zinc-finger antiviral protein acts as a tumor suppressor in colorectal cancer. Oncogene.

[160] Du Y., Carling T., Fang W., Piao Z., Sheu J.C., Huang S. (2001). Hypermethylation in human cancers of the RIZ1 tumor suppressor gene, a member of a histone/protein methyltransferase superfamily. Cancer Res..

[161] Jiang G.L., Huang S. (2001). Adenovirus expressing RIZ1 in tumor suppressor gene therapy of microsatellite-unstable colorectal cancers. Cancer Res..

[162] Barz T., Hoffmann A., Panhuysen M., Spengler D. (2006). Peroxisome proliferator-activated receptor gamma is a Zac target gene mediating Zac antiproliferation. Cancer Res..

[163] Kowalczyk A.E., Krazinski B.E., Godlewski J., Kiewisz J., Kwiatkowski P., Sliwinska-Jewsiewicka A., Kiezun J., Wierzbicki P.M., Bodek G., Sulik M. (2015). Altered expression of the PLAGL1 (ZAC1/LOT1) gene in colorectal cancer: Correlations to the clinicopathological parameters. Int. J. Oncol..

[164] Brown A.R., Simmen R.C., Raj V.R., Van T.T., MacLeod S.L., Simmen F.A. (2015). Kruppel-like factor 9 (KLF9) prevents colorectal cancer through inhibition of interferon-related signaling. Carcinogenesis.

[165] Jiang J., Liu L.Y. (2015). Zinc finger protein X-linked is overexpressed in colorectal cancer and is associated with poor prognosis. Oncol. Lett..

[166] Yan X., Shan Z., Yan L., Zhu Q., Liu L., Xu B., Liu S., Jin Z., Gao Y. (2016). High expression of Zinc-finger protein X-linked promotes tumor growth and predicts a poor outcome for stage II/III colorectal cancer patients. Oncotarget.

[167] Yan X., Yan L., Su Z., Zhu Q., Liu S., Jin Z., Wang Y. (2014). Zinc-finger protein X-linked is a novel predictor of prognosis in patients with colorectal cancer. Int. J. Clin. Exp. Pathol..

[168] Jung J.H., Jung D.B., Kim H., Lee H., Kang S.E., Srivastava S.K., Yun M., Kim S.H. (2018). Zinc finger protein 746 promotes colorectal cancer progression via c-Myc stability mediated by glycogen synthase kinase 3beta and F-box and WD repeat domain-containing 7. Oncogene.

[169] Chen J., Tang H., Wu Z., Zhou C., Jiang T., Xue Y., Huang G., Yan D., Peng Z. (2013). Overexpression of RBBP6, alone or combined with mutant TP53, is predictive of poor prognosis in colon cancer. PLoS ONE.

[170] Tang Y.A., Chen Y.F., Bao Y., Mahara S., Yatim S., Oguz G., Lee P.L., Feng M., Cai Y., Tan E.Y. (2018). Hypoxic tumor microenvironment activates GLI2 via HIF-1alpha and TGF-beta2 to promote chemoresistance in colorectal cancer. Proc. Natl. Acad. Sci. USA.

[171] Yao J., Lei P.J., Li Q.L., Chen J., Tang S.B., Xiao Q., Lin X., Wang X., Li L.Y., Wu M. (2020). GLIS2 promotes colorectal cancer through repressing enhancer activation. Oncogenesis.

[172] Chen M.S., Lo Y.H., Chen X., Williams C.S., Donnelly J.M., Criss Z.K., Patel S., Butkus J.M., Dubrulle J., Finegold M.J. (2019). Growth Factor-Independent 1 Is a Tumor Suppressor Gene in Colorectal Cancer. Mol. Cancer Res..

[173] Jones C., St-Jean S., Frechette I., Bergeron D., Rivard N., Boudreau F. (2013). Identification of a novel promyelocytic leukemia zinc-finger isoform required for colorectal cancer cell growth and survival. Int. J. Cancer.

[174] Wang D.Q., Wang K., Yan D.W., Liu J., Wang B., Li M.X., Wang X.W., Liu J., Peng Z.H., Li G.X. (2014). Ciz1 is a novel predictor of survival in human colon cancer. Exp. Biol. Med..

[175] Yin J., Wang C., Tang X., Sun H., Shao Q., Yang X., Qu X. (2013). CIZ1 regulates the proliferation, cycle distribution and colony formation of RKO human colorectal cancer cells. Mol. Med. Rep..

[176] Thoma O.M., Neurath M.F., Waldner M.J. (2021). Cyclin-Dependent Kinase Inhibitors and Their Therapeutic Potential in Colorectal Cancer Treatment. Front. Pharmacol..

[177] Aponte P.M., Caicedo A. (2017). Stemness in Cancer: Stem Cells, Cancer Stem Cells, and Their Microenvironment. Stem Cells Int..

[178] Jian Y., Wang M., Zhang Y., Ou R., Zhu Z., Ou Y., Chen X., Liang X., Ding Y., Song L. (2018). Jade family PHD finger 3 (JADE3) increases cancer stem cell-like properties and tumorigenicity in colon cancer. Cancer Lett..

[179] Kim J., Moon Y. (2021). Mucosal ribosomal stress-induced PRDM1 promotes chemoresistance via stemness regulation. Commun. Biol..

[180] Sun B., Xu L., Bi W., Ou W.B. (2022). SALL4 Oncogenic Function in Cancers: Mechanisms and Therapeutic Relevance. Int. J. Mol. Sci..

[181] Cheng J., Deng R., Wu C., Zhang P., Wu K., Shi L., Liu X., Bai J., Deng M., Gao J. (2015). Inhibition of SALL4 suppresses carcinogenesis of colorectal cancer via regulating Gli1 expression. Int. J. Clin. Exp. Pathol..

[182] Hao L., Zhao Y., Wang Z., Yin H., Zhang X., He T., Song S., Sun S., Wang B., Li Z. (2016). Expression and clinical significance of SALL4 and beta-catenin in colorectal cancer. J. Mol. Histol..

[183] Xue W., Wang F., Han P., Liu Y., Zhang B., Gu X., Wang Y., Li M., Zhao Y., Cui B. (2020). The oncogenic role of LncRNA FAM83C-AS1 in colorectal cancer development by epigenetically inhibits SEMA3F via stabilizing EZH2. Aging.

[184] Zhu C., Zhang L., Zhao S., Dai W., Xu Y., Zhang Y., Zheng H., Sheng W., Xu Y. (2021). UPF1 promotes chemoresistance to oxaliplatin through regulation of TOP2A activity and maintenance of stemness in colorectal cancer. Cell Death Dis..

[185] Alvarez C., Quiroz A., Benitez-Riquelme D., Riffo E., Castro A.F., Pincheira R. (2021). SALL Proteins; Common and Antagonistic Roles in Cancer. Cancers.

[186] Kim S.T., Sohn I., Do I.G., Jang J., Kim S.H., Jung I.H., Park J.O., Park Y.S., Talasaz A., Lee J. (2014). Transcriptome analysis of CD133-positive stem cells and prognostic value of survivin in colorectal cancer. Cancer Genom. Proteom..

[187] Igarashi H., Taniguchi H., Nosho K., Ishigami K., Koide H., Mitsuhashi K., Okita K., Takemasa I., Imai K., Nakase H. (2020). PRDM14 promotes malignant phenotype and correlates with poor prognosis in colorectal cancer. Clin. Transl. Oncol..

[188] Liu M.L., Zang F., Zhang S.J. (2019). RBCK1 contributes to chemoresistance and stemness in colorectal cancer (CRC). Biomed. Pharmacother..

[189] Cassandri M., Smirnov A., Novelli F., Pitolli C., Agostini M., Malewicz M., Melino G., Raschella G. (2017). Zinc-finger proteins in health and disease. Cell Death Discov..

[190] Fedotova A.A., Bonchuk A.N., Mogila V.A., Georgiev P.G. (2017). C2H2 Zinc Finger Proteins: The Largest but Poorly Explored Family of Higher Eukaryotic Transcription Factors. Acta Nat..

[191] Munro D., Ghersi D., Singh M. (2018). Two critical positions in zinc finger domains are heavily mutated in three human cancer types. PLoS Comput. Biol..

[192] Abbehausen C. (2019). Zinc finger domains as therapeutic targets for metal-based compounds—An update. Metallomics.

[193] Yan D., Aiba I., Chen H.H., Kuo M.T. (2016). Effects of Cu(II) and cisplatin on the stability of Specific protein 1 (Sp1)-DNA binding: Insights into the regulation of copper homeostasis and platinum drug transport. J. Inorg. Biochem..

[194] Shimberg G.D., Ok K., Neu H.M., Splan K.E., Michel S.L.J. (2017). Cu(I) Disrupts the Structure and Function of the Nonclassical Zinc Finger Protein Tristetraprolin (TTP). Inorg. Chem..

[195] Sievers Q.L., Petzold G., Bunker R.D., Renneville A., Slabicki M., Liddicoat B.J., Abdulrahman W., Mikkelsen T., Ebert B.L., Thoma N.H. (2018). Defining the human C2H2 zinc finger degrome targeted by thalidomide analogs through CRBN. Science.

[196] Ma T., Shi S., Jiang H., Chen X., Xu D., Ding X., Zhang H., Xi Y. (2021). A pan-cancer study of spalt-like transcription factors 1/2/3/4 as therapeutic targets. Arch. Biochem. Biophys..

[197] Zhao Z., Wang L., Bartom E., Marshall S., Rendleman E., Ryan C., Shilati A., Savas J., Chandel N., Shilatifard A. (2019). beta-Catenin/Tcf7l2-dependent transcriptional regulation of GLUT1 gene expression by Zic family proteins in colon cancer. Sci. Adv..

[198] Satow R., Inagaki S., Kato C., Shimozawa M., Fukami K. (2017). Identification of zinc finger protein of the cerebellum 5 as a survival factor of prostate and colorectal cancer cells. Cancer Sci..

[199] Liu Y., Huang W., Gao X., Kuang F. (2019). Regulation between two alternative splicing isoforms ZNF148(FL) and ZNF148(DeltaN), and their roles in the apoptosis and invasion of colorectal cancer. Pathol. Res. Pract..

[200] Essien B.E., Sundaresan S., Ocadiz-Ruiz R., Chavis A., Tsao A.C., Tessier A.J., Hayes M.M., Photenhauer A., Saqui-Salces M., Kang A.J. (2016). Transcription Factor ZBP-89 Drives a Feedforward Loop of beta-Catenin Expression in Colorectal Cancer. Cancer Res..

[201] Gao X.H., Liu Q.Z., Chang W., Xu X.D., Du Y., Han Y., Liu Y., Yu Z.Q., Zuo Z.G., Xing J.J. (2013). Expression of ZNF148 in different developing stages of colorectal cancer and its prognostic value: A large Chinese study based on tissue microarray. Cancer.

[202] Xia L., Lin H., Zhou Y., Lian J. (2022). ZNF750 facilitates carcinogenesis via promoting the expression of long non-coding RNA CYTOR and influences pharmacotherapy response in colon adenocarcinoma. J. Zhejiang Univ. Sci. B.

[203] Qu J., Zhang X., Lv X. (2020). Zinc finger protein 750(ZNF750), negatively regulated by miR-17-5p, inhibits proliferation, motility and invasion of colonic cancer cells. J. Gene Med..

[204] Jiang X., Yang Z., Li Z. (2019). Zinc finger antisense 1: A long noncoding RNA with complex roles in human cancers. Gene.

[205] Guo X., Jing Y.M., Lou H.Z., Lou Q.A. (2019). Effect and mechanism of long non-coding RNA ZEB2-AS1 in the occurrence and development of colon cancer. Math Biosci. Eng..

[206] Jin Z., Chen B. (2020). LncRNA ZEB1-AS1 Regulates Colorectal Cancer Cells by MiR-205/YAP1 Axis. Open. Med..

